# Evaluating the strengths and weaknesses of large language models in answering neurophysiology questions

**DOI:** 10.1038/s41598-024-60405-y

**Published:** 2024-05-11

**Authors:** Hassan Shojaee-Mend, Reza Mohebbati, Mostafa Amiri, Alireza Atarodi

**Affiliations:** 1 Department of General Courses, Faculty of Medicine, Gonabad University of Medical Sciences, Gonabad, Iran; 2 Department of Physiology, Faculty of Medicine, Gonabad University of Medical Sciences, Gonabad, Iran; 3 Department of English Language and General Courses, School of Medicine, Mashhad University of Medical Sciences, Mashhad, Iran; 4 Department of Knowledge and Information Science, Paramedical College and Social Development & Health Promotion Research Center, Gonabad University of Medical Sciences, Gonabad, Iran

**Keywords:** Large language models, Neurophysiology, Evaluation, Bloom’s taxonomy, Physiology, Neurophysiology

## Abstract

Large language models (LLMs), like ChatGPT, Google’s Bard, and Anthropic’s Claude, showcase remarkable natural language processing capabilities. Evaluating their proficiency in specialized domains such as neurophysiology is crucial in understanding their utility in research, education, and clinical applications. This study aims to assess and compare the effectiveness of Large Language Models (LLMs) in answering neurophysiology questions in both English and Persian (Farsi) covering a range of topics and cognitive levels. Twenty questions covering four topics (general, sensory system, motor system, and integrative) and two cognitive levels (lower-order and higher-order) were posed to the LLMs. Physiologists scored the essay-style answers on a scale of 0–5 points. Statistical analysis compared the scores across different levels such as model, language, topic, and cognitive levels. Performing qualitative analysis identified reasoning gaps. In general, the models demonstrated good performance (mean score = 3.87/5), with no significant difference between language or cognitive levels. The performance was the strongest in the motor system (mean = 4.41) while the weakest was observed in integrative topics (mean = 3.35). Detailed qualitative analysis uncovered deficiencies in reasoning, discerning priorities, and knowledge integrating. This study offers valuable insights into LLMs’ capabilities and limitations in the field of neurophysiology. The models demonstrate proficiency in general questions but face challenges in advanced reasoning and knowledge integration. Targeted training could address gaps in knowledge and causal reasoning. As LLMs evolve, rigorous domain-specific assessments will be crucial for evaluating advancements in their performance.

## Introduction

The world is currently experiencing significant transformations as new tools and technology permeating every corner and aspect of our lives. People are shocked, contemplating the pros, cons and wondering how these advancements will impact us. Can we rely on these innovations? To find answers, researchers are delving into various approaches. They enter artificial intelligence (AI), a captivating and significant phenomenon of our time, with versatile capabilities applicable to a wide range of tasks. Recently, there have been remarkable advancements in natural language processing (NLP). This progress has given rise to sophisticated large language models (LLMs) that can engage with humans in a remarkably human-like manner. Specifically, chatbot platforms have made strides, providing accurate and contextually appropriate responses to users’ queries^1^. With this ongoing progress, there is a growing demand for reliable and efficient question-answering systems in specialized domains like neurophysiology.

The rapid advancements in conversational AI have given rise to advanced language models capable of generating humanlike writing. With their wide range of functionalities, including generating human-like responses, proficiency in professional exams, complex problem-solving, and more, these models have captivated interest^2^. Large language models (LLMs) are becoming increasingly popular in both academia and industry owing to their unprecedented performance in various applications. As LLMs continue to play a vital role in both research and everyday activities, their evaluation becomes increasingly critical, not only at the task level but also at the society level to better comprehend their potential risks. In recent years, substantial efforts have been devoted to examine LLMs from diverse perspectives^3^.

With the popularization of software like OpenAI’s ChatGPT, Google’s Bard and Anthropic’s Claude, LLMs have permeated various aspects of life and work. They are used to provide customized recipes, suggesting substitutions for missing ingredients. It can be used to draft research proposals, write working code in many programming languages, translate text between languages, assist in policy making, and more. Users interact with LLMs through “prompts” or natural language instructions. Carefully designed prompts can significantly enhance the quality of better outputs^4^. These models, designed to emulate human intelligence, employ statistical analyses to understand patterns and connections among words and phrases^1^.

Neurophysiology, a key branch of neuroscience, is dedicated to unraveling the complex mechanisms governing the nervous system's operations. Investigating neurophysiological phenomena necessitates a deep grasp of diverse concepts, theories, and experimental approaches. Consequently, having a highly competent question-answering system capable of addressing neurophysiology inquiries is of utmost importance to researchers, clinicians, and students in this field. Questions in the system can be categorized into two categories, lower-order and higher-order questions, aligned with Bloom's taxonomy, enabling the assessment of language models' ability to respond to queries in each category. Bloom's taxonomy, a widely utilized framework in educational contexts, classifies cognitive levels into six domains: knowledge, comprehension, application, analysis, synthesis, and evaluation^5^. By applying Bloom’s taxonomy to evaluate LLMs, their efficacy in answering questions spanning various cognitive levels, including those in neurophysiology, can be gauged^6^. By considering how well ChatGPT, Bard, and Claude perform at different topics and different levels of Bloom's taxonomy, their abilities to comprehensively and accurately address neurophysiology questions.

Previous publications evaluating LLMs across various disciplines have covered fields such as, gastroenterology^7^, pathology^8^, neurology^9^, physiology^6,10^, and solving case vignettes in physiology^11^. In a cross-sectional study, the performance of LLMs on neurology board–style examinations were assessed using a question bank approved by the American Board of Psychiatry and Neurology. The questions were categorized into lower-order and higher-order based on the Bloom taxonomy for learning and assessment^9^. To the best of our knowledge there was no study specifically on evaluating LLMs in the field of neurophysiology. Additionally, in studies within similar domains, most studies have investigated the ability of LLMs to provide accurate answers for multiple-choice questions^12–14^. To comprehensively understand the strengths and weaknesses of these models in a sophisticated field like neurophysiology, it is essential to evaluate the capabilities of these models in responding to essay questions, across all cognitive levels. Neurophysiology presents a diverse range of questions levels, making it a valuable area for assessing the strengths and limitations of LLMs.

This study compares the performance of three language models, namely, ChatGPT, Bard, and Claude, in answering neurophysiology questions in both the Persian and English languages. It focuses on various cognitive levels based on Bloom's Taxonomy and evaluates the models' reasoning process by asking for the rationale behind their responses. The study aims to evaluate the performance of the LLMs in addressing neurophysiology questions in different cognitive levels, along with determining whether the models rely on memorization or demonstrate analytical reasoning and logical explanations. Moreover, it offers insights into the capabilities of the LLMs by identifying potential reasons for incorrect answers to determine their weaknesses in responding to neurophysiology questions.

## Methodology

This exploratory, applicational and cross-sectional study was carried out using AI-driven chat applications, including ChatGPT (chat.openai.com), Claude (claude.ai), and Bard (bard.google.com), which offer free services for researchers. The researchers aimed to assess the strengths and weaknesses of the selected LLMs in their ability to answer neurophysiology questions.

### Questions

A total of 20 questions were chosen from four topics in neurophysiology, including general, sensory, motor, and integrative systems, with each topic comprising 5 questions. The LLMs were asked to provide explanations for their selected answers for all questions, which encompassed true/false, multiple-choice, and essay formats. Therefore, all the questions were effectively essay questions allowing for a scoring range of 0–5 points for the responses. Furthermore, the questions were categorized based on cognitive skills into lower-order and higher-order categories, with each topic included 3 lower-order and 2 higher-order questions.

It is worth noting that, according to Bloom’s taxonomy, memorization and recall are categorized as lower-level cognitive skills, necessitating only a minimal degree of comprehension. In contrast, the application of knowledge and critical thinking fall under the category of higher-level cognitive skills, requiring deep conceptual understating^15^. A panel of three skilled physiologists was chosen to validate the questions and evaluate the answers of the LLMs to the questions. They were university lecturers who had at least 2 years of teaching experience in neurophysiology to medical students. The questions, topics, and cognitive skills are listed in Table 1 of Supplementary 1.

### Data collection

The latest versions of ChatGPT 3.5 (November 21, 2023), Claude 2 (December 5, 2023), and Bard (November 21, 2023) were prompted with questions in both Persian and English languages. These versions are undergoing public testing for academic research. The Persian and English questions, along with the answers generated by the three selected LLMs, were stored in separate files for evaluation by the physiologists.

Notably, prompt engineering is essential to improve the efficiency of LLMs. It includes strategies such as chain-of-thought (CoT) prompting and structured prompting^16^. The CoT prompting has achieved the state-of-the-art performances in arithmetic and symbolic reasoning^17,18^. The model is instructed in the CoT prompting to provide step-by-step reasoning in generating a final answer, which could be few-shot or zero-shot^19^. Utilizing structured prompting, which includes important components such as context, the expected behavior, and the format of the output, is another strategy for achieving optimal outcomes. In this study, zero-shot CoT was employed by adding "let's think step by step" into the questions. Also, the following structured prompt was used for all the questions: “Imagine you are an expert physiologist with a specializing in neurophysiology. Answer the following question. {question…}. Explain the steps and reasons that lead you to the answer. write your final answer. Let’s think step by step”.

The panel of three physiologists was asked to score each question on a scale of 0 to 5 points, where a score of 5 indicated a full and comprehensive response to the question. All data were recorded in an Excel file for further analysis.

### Statistical analysis

The statistical analysis employed mean, median and standard deviation to provide a comprehensive overview of the data. The Friedman test was used to assess if there were statistically significant variations in the scores of LLMs between Persian and English languages, with each group comprising 20 questions. Furthermore, the Kruskal‒Wallis’s test was carried out to assess the significance of score differences across four topics and two levels of cognitive skills. The intraclass correlation coefficient (ICC), a two-way random model with absolute agreement,^20^ was used to evaluate the level of agreement among the physiologists' scores. Furthermore, the Wilcoxon signed rank test was applied to ascertain the significant difference between the scores of LLMs in Persian and English. A p value of below 0.05 was considered statistically significant. All statistical analyses were performed using SPSS software, version 22.

## Results

The responses from three LLMs, ChatGPT, Bard, and Claude, were collected for both Persian and English languages questions. Three experienced physiologists evaluated the responses. Each question was given to the LLMs only once, simulating a student answering neurophysiology questions in an exam setting. As a result, the ambiguity of the questions or the LLMs lack of understanding of the question content or the unimportant content that should not be mentioned in the responses could affect the scores that the LLMs received from each question. The Persian questions along with the answers of LLMs to these questions are shown in Supplementary 2, while the English questions along with the answers of LLMs are shown in Supplementary 3 and the evaluation results from the experts, including the average scores they assigned, are summarized in Supplementary 1 Table 2.

The evaluation results using by ICC, showed good agreement among the physiologists in scoring. The ICC values for various topics ranged from 0.935 to 0.993. The ICC value for all questions was 0.978 (F = 51.217, p < 0.001). This high level of agreement in the physiologists' scores signifies the reliability of expert opinions. The results of the ICC test among the physiologists are shown in Table 1.

**Table 1:** Interrater agreement among physiologists.

Topic	N	Intraclass correlation	95% Confidence interval		Sig
			Lower bound	Upper bound	
General	30	0.992	0.984	0.996	< 0.001
Sensory system	30	0.985	0.970	0.992	< 0.001
Motor system	30	0.962	0.931	0.981	< 0.001
Integrative	30	0.957	0.886	0.982	< 0.001
Total	120	0.978	0.969	0.985	< 0.001

**Figure 1:**
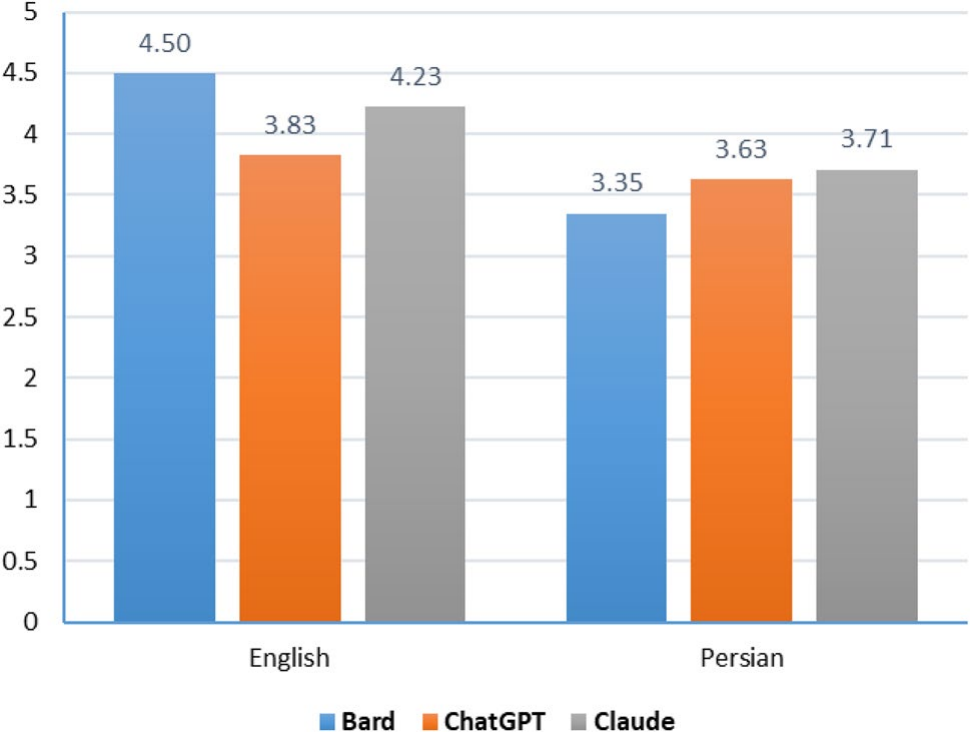
Mean scores for all LLMs in Persian and English.

Given the good agreement between the raters, the mean of their scores was used as the score for each question in the subsequent analysis. The evaluation results from the physiologists showed that the overall performance of selected LLMs in responding to the questions, as well as the performance of each of LLMs in both English and Persian languages, were deemed satisfactory (Table 2). The overall mean score obtained for the questions was 3.87 ± 1.7. As illustrated in Fig. 1, the mean scores for various LLMs in the Persian and English languages ranged from 3.35 (Bard in Persian) to 4.50 (Bard in English). Nevertheless, the results of the Friedman test did not reveal any statistically significant difference in LLMs scores between Persian (p = 0.794) and English (p = 0.281). Overall, the average scores in English (Mean = 4.18, Median = 4.64) surpassed those in Persian (Mean = 3.56, Median = 4.72). However, the Wilcoxon signed rank test showed that this difference was not statistically significant (p = 0.222).

**Table 2:** Mean ± SD (Median) of scores by language, topic, and LLM.

		General	Sensory system	Motor system	Integrative	Total
English	Bard	4.9 ± 0.22 (5)	4.67 ± 0.75 (5)	4.83 ± 0.37 (5)	3.6 ± 1.65 (4.33)	4.5 ± 1.01 (5)
	ChatGPT	4.13 ± 1.94 (5)	4.47 ± 0.69 (4.67)	3.87 ± 2.02 (5)	2.83 ± 1.38 (2.33)	3.83 ± 1.6 (4.83)
	Claude	4.93 ± 0.15 (5)	4.5 ± 0.8 (5)	3.3 ± 1.95 (3.83)	4.17 ± 1.44 (5)	4.23 ± 1.32 (5)
Persian	Bard	4 ± 2.24 (5)	1.2 ± 2.17 (0)	4.8 ± 0.3 (5)	3.4 ± 1.92 (3.67)	3.35 ± 2.17 (4.83)
	ChatGPT	4 ± 2.24 (5)	2.7 ± 2.43 (3.17)	4.87 ± 0.3 (5)	2.93 ± 1.69 (3.33)	3.63 ± 1.93 (5)
	Claude	4.2 ± 1.79 (5)	2.67 ± 2.48 (3.67)	4.8 ± 0.18 (4.67)	3.17 ± 1.72 (3.5)	3.71 ± 1.83 (4.67)
Total		4.36 ± 1.59 (5)	3.37 ± 2.06 (4.5)	4.41 ± 1.23 (5)	3.35 ± 1.56 (3.58)	3.87 ± 1.7 (5)

**Figure 2:**
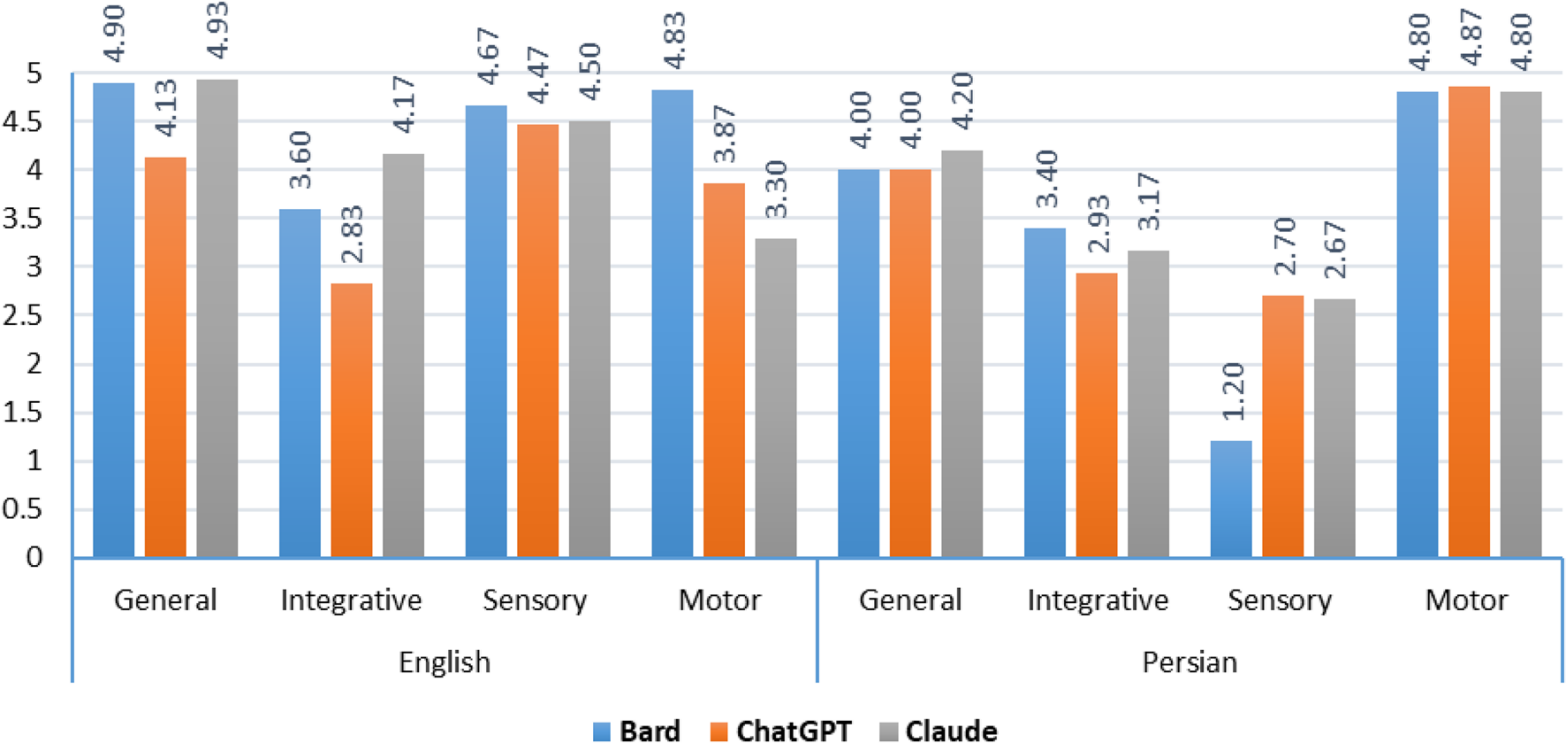
Mean scores for LLMs in each topic and language.

Regarding different topics, the highest scores were associated with the motor system topic, while the lowest score was obtained for the integrative topic (Table 2). Based on the results, the performance of LLMs can be generally evaluated as excellent for general and motor system topics, good for sensory system and integrative topics. The best scores for the English questions were attributed to the general topic, whereas the weakest scores for the Persian questions were linked to the sensory topic (Fig. 2). The results of the Kruskal‒Wallis’s test revealed a significant difference in the scores for the integrative topic compared to other topics (p < 0.001).

**Figure 3:**
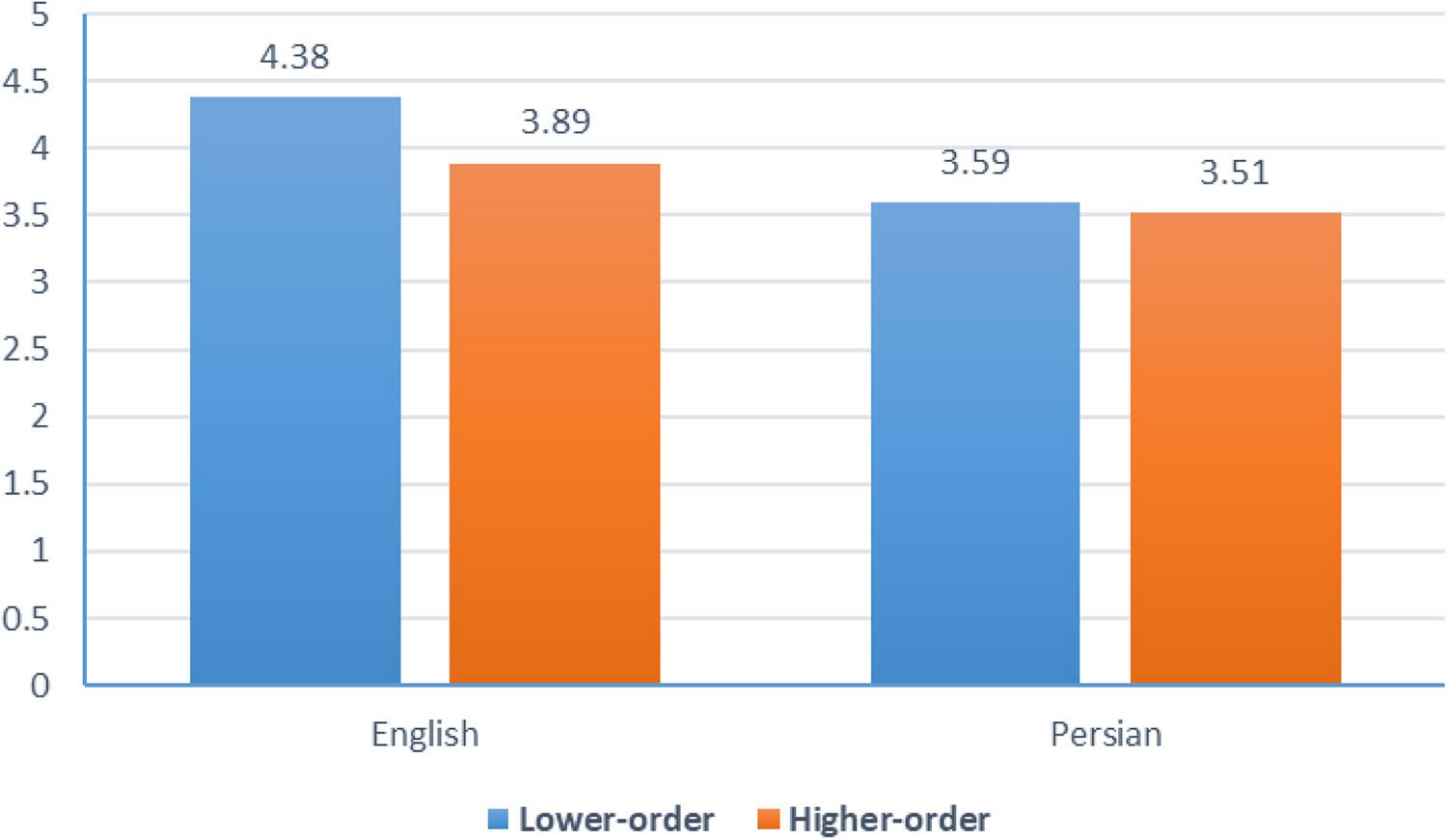
Mean scores for cognitive skills in Persian and English.

Moreover, regarding the cognitive level of the questions, the results of the Kruskal‒Wallis’s test indicated that there was no significant difference between the scores (p = 0.613). The lowest score of 3.51 was recorded for higher-order questions in Persian, while the highest score of 4.38 was achieved for lower-order questions in English (Fig. 3).

**Figure 4:**
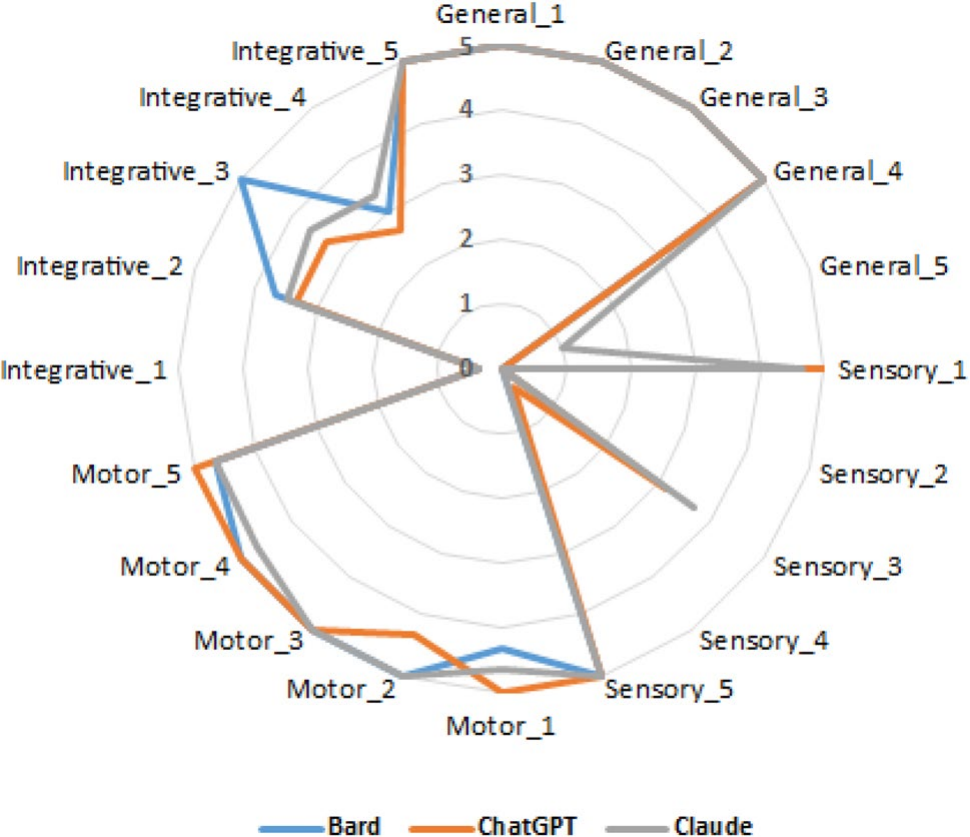
Scores of LLMs to Persian questions.

**Figure 5:**
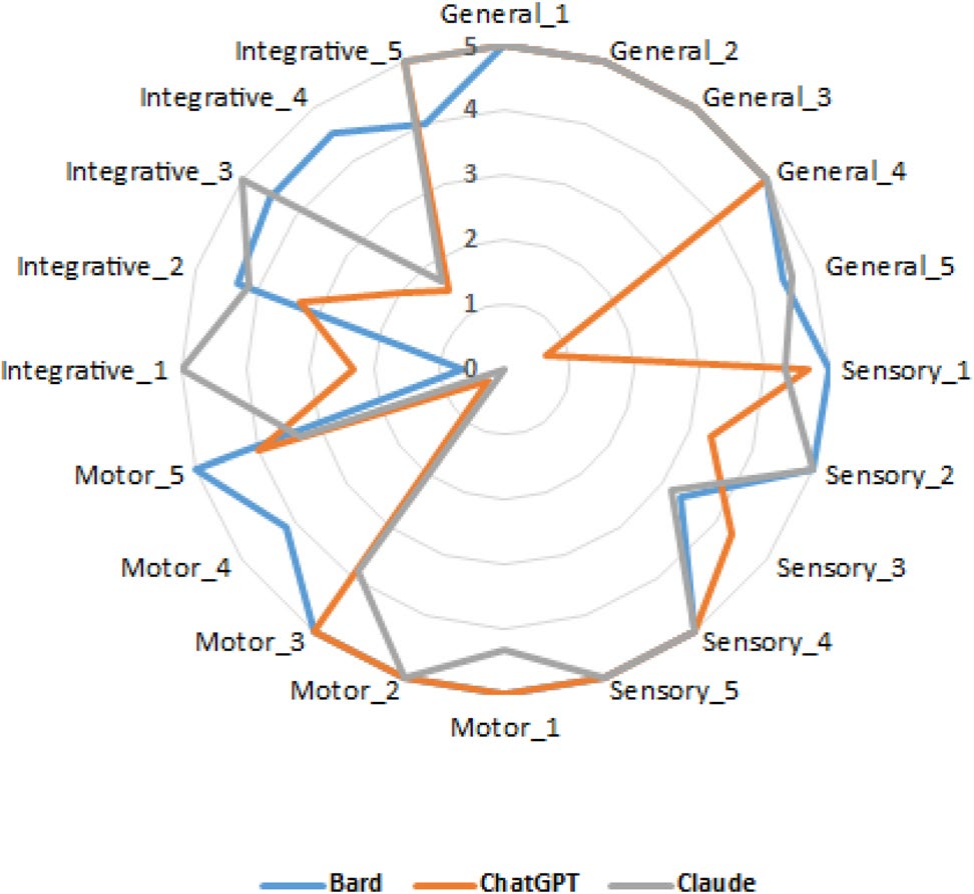
Scores of LLMs to English questions.

Figures 4 and 5 show the mean scores for different questions in the Persian and English languages. The proximity of the curves indicates the similarity in scores in different LLMs, while the closer the curves are to the outer edge of the diagram signifies higher scores for those question. The diagrams suggest that for most questions, there is a comparable performance level among different LLMs. However, this consistency is not observed for certain questions. For instance, in the Persian questions, for the Sensory_1 question, ChatGPT and Claude were provided nearly complete answers, but Bard received a score of zero. In addition, for Sensory_3, the scores of ChatGPT and Claude achieved fairly scores, while Bard was unable to answer the question. In contrast, for Integrative_3, both ChatGPT and Claude were unable to provide an answer, but Bard managed to receive a perfect score for the question (Fig. 4).

For English questions, there are also questions where there is no similarity in performance among LLMs. For example, both Bard and Claude received almost full scores for General_5, but ChatGPT struggled to provide correct answers to these questions. Moreover, for Motor_4, both ChatGPT and Claude were unable to offer a satisfactory response, whereas Bard's answer was almost complete. In contrast, for Integrative_4, both ChatGPT and Claude fell short in providing a good answer, but Bard managed to achieve a perfect score for the question (Fig. 5).

In addition to the inconsistency in responses, in some questions, almost none of the LLMs were able to adequately respond to the question. For further analysis, the questions to which LLMs couldn’t respond adequately were identified. The total possible scores of the three language models for each question in Persian and English were 15. Questions with a mean score of 3 or less for each LLM were selected based on the criterion. Therefore, questions for which the total score of all LLMs were equal or less than 9 were chosen. In Persian, the selected questions included General_5, Sensory_2, Sensory_3, Sensory_4, Integrative_1 and Integrative_4 questions. Additionally, for the English questions, the total score was below 9 for Motor_4, Integrative_1 and Integrative_4.

### General_5 question: Is myelination of postganglionic sympathetic fibers done by Schwann cells?

The correct answer to this question is that postganglionic sympathetic fibers lack myelin. The use of the phrase “by Schwann cells” in the question stem is a misleading phrase. In the Persian language, none of the language models could provide the correct answer even after removing the misleading phrase from the question. Through further questions, it became clear that in Persian, postganglionic sympathetic fibers were incorrectly categorized as type A instead of type C. Also, none of the models had sufficient information regarding which types of fibers are myelinated. Hence, the cause of the wrong answer in the Persian language can be considered as "having inaccurate information" in the LLMs, but by removing the misleading phrase from the question, all LLMs were able to provide the correct answer in the English language. Therefore, the cause of the initial incorrect answer in English in the ChatGPT can be attributed to the presence of a “misleading phrase in the question”.

### Sensory_2 question: Are sexual sensations mostly transmitted through the posterior column—medial lemniscus?

The correct answer to this question is “No”. In Persian, Bard did not provide a response to the question and instead wrote: “I am a language model and do not have the capacity to understand or respond to this query”. Probably the Persian equivalent of the phrase “sexual sensations” has led to this response. Two other LLMs also failed to provide a correct response. By changing the question and using the English phrase equivalent to the ‘posterior column-medial lemniscus’ in Persian all LLMs were able to provide the correct answer in Persian. Therefore, the reason for the wrong answer to this question in Persian can be expressed as “incorrect translation for phrases in Persian”.

### Sensory_3 question: State key components, including nuclei and neurotransmitters, in the central nervous system analgesic pathway?

The correct answer is “the PAG projects enkephalinergic neurons to the Raphe, and after stimulation, the serotonergic projections go to the spine and stimulate the enkephalinergic neurons that cause pain inhibition”. In response to this essay question, the LLMs failed to mention some important nuclei or mentioned nuclei that were of lesser importance. This means that the most important phrase in the question was not considered. This lack of attention to importance was present in both the Persian and English languages responses, with a more pronounced effect in Persian. Thus, the reason for the incorrect response to this question can be stated as “not considering the importance and priority” and providing “insignificant additional explanation” compared to a knowledgeable individual in this field.

### Sensory_4 question: Which sensation is NOT transmitted through the anterolateral pathway? A) Chronic pain B) Cold sensation C) Touch sensation from Meissner receptor D) Touch sensation from Ruffini receptors.

The sensation that is not transmitted through the anterolateral pathway is (C) Touch sensation from Meissner receptors. LLMs in English provided the correct answer to this question, whereas LLMs in Persian answered it incorrectly. Claude stated that Meissner receptors transmit the sensation of pressure to the brain, while Ruffini receptors transmit the sensations of contact and vibration. However, the opposite of this statement is correct. Moreover, ChatGPT and Bard offered general rather than specialized information with details regards to this question. Hence, the reason for the incorrect response in the Persian language can be attributed to as “inaccurate information” and “insufficient specialized knowledge” in Persian language concerning this question.

### Motor_4 question: Does microinjection of glutamate into the medullary reticular nucleus cause relaxation of axial muscles?

The correct answer is “Yes”. ChatGPT and Claude failed to provide the accurate response to this question. Research indicates that neural projections can exhibit both excitatory and inhibitory functions. So, these two LLMs focused on the excitatory aspect. However, stronger evidence from textbooks supports the idea that neural projections can indeed be inhibitory. Therefore, the reason for the incorrect response can be attributed to “neglecting the significance of available evidence”.

### Integrative_1 question: In medical science and neurophysiology, is knowing “my birthday is January 10, 1998” an example of semantic explicit memory?

The correct answer is “No”. Because stating my birthday date is only a claim about a past event, which can be considered a verified fact if supported by evidence confirming that event. None of the LLMs, except for Claude, managed to provide the correct response in either Persian or English. They mistakenly treated this statement as a fact.

Most likely, the reason for that is the absence of a similar sentence in the training texts used for the LLMs. Therefore, the reason for the incorrect answer to this question can be considered “using non-existent example” and “lack of reasoning ability” for questions that require reasoning based on prior knowledge and applying that knowledge to the current context.

### Integrative_4 question: In medical science and neurophysiology, which of the following represents explicit memory? A) The Shahnameh is the masterpiece of the great Iranian poet named Ferdowsi B) Today I arrived about 7 minutes late to physiology class. I'm usually late for classes. C) In 2010 my house had a major fire D) One of my elementary school friends’ last names ended in “Abadi” or “Abadian”

The correct answers are A and C. ChatGPT correctly identified that option A is a fact and pertains to semantic memory. Also, it initially stated that the explicit memory consists of semantic and episodic types. However, in the final summary, despite initially identifying it as semantic, it failed to categorize it as explicit memory.

Regarding option B, it also correctly mentioned that it does not pertain to long-term memory and therefore, cannot be explicit memory. Yet in the final summary, it categorized it as explicit memory. For option D, the lack of accurate recollection of the past, a complete memory has not formed and therefore it is not explicit, which most LLMs failed to identify. Therefore, the reason for the incorrect answer can be considered as “insufficient specialized information” and “lack of reasoning ability”. The facts are correctly stated step-by-step, but combining these facts and deducing conclusions from them is not executed effectively.

## Discussion

Three LLMs, ChatGPT, Bard, and Claude, were used to assess their capacity in providing comprehensive and logical answers to neurophysiology essay prompts in both Persian and English languages. These LLMs can respond to complex commands by analyzing and comprehending the supplied text, utilizing their highly advanced natural language processing capabilities and their vast training datasets^8^. The results showed that, overall, the models demonstrated commendable proficiency in addressing neurophysiology queries. However, certain variations among the models were observed depending upon the specific topic of the inquiries.

Across the various topics analyzed, the LLMs performed the best on queries concerning to the motor system and general neurophysiology, indicating their strength in addressing fundamental principles. In terms of sensory system topics, the performance was moderately solid, suggesting that the models can comprehend and explain sensory neurophysiology to a certain degree. However, when faced with integrative questions, the scores significantly dropped. This underscores a present constraint of the models in tackling complex, multi-step reasoning requiring integration of knowledge across neurophysiology topics. Tailored training focusing on integrative concepts could help improve LLMs’ capabilities in this realm ^17^.

Interestingly, although there were no significant disparities in the performance of the models in Persian and English or between lower-order and higher-order questions, a detailed analysis revealed some inconsistencies. A qualitative analysis of the responses unveiled deficiencies in reasoning capabilities, particularly evident in unfamiliar question scenarios that necessitate adaptable application of knowledge. For certain questions, one model excelled, while others faltered, without a discernible pattern. This lack of uniformity implies knowledge gaps and variances in the training of the distinct models^21^. Additionally, all three models struggled with several complex questions in both languages, yielding subpar scores. This further underscore the limitations of these models in advanced reasoning and handling ambiguous and multifaceted questions.

When comparing languages, the scores were mostly comparable for all the LLMs. The models appeared to have acquired sufficient linguistic knowledge proficiency to comprehend and provide accurate responses in both languages. Nonetheless, a few incorrect answers unique to Persian emphasized deficiencies in the information encoded in the models for that language. Overall, the outcomes confirm the effectiveness of LLMs for addressing neurophysiology inquiries in various languages.

An in-depth review of the incorrect responses shed light on the specific limitations of the LLMs. Providing flawed information and the inability to discern key aspects of questions emerged as some of the deficiencies. However, some studies have reported a satisfactory reasoning level in LLMs^22^, and a deficiency in reasoning for unfamiliar scenarios has been identified as one of the deficiencies in providing correct answers in various questions. These gaps need to be addressed through more extensive training of the models utilizing high-quality data encompassing diverse neurophysiology topics, contexts, and linguistic nuances. The subpar performance on integrative questions can be attributed to the models' reliance on memorization and pattern recognition from the training data rather than a profound comprehension of the concepts.

Although large datasets help them to remember facts and terminology, it is still difficult for LLMs to integrate knowledge across topics to solve new problems. Although previous studies demonstrating that CoT prompting improves the reasoning abilities of the LLMs^16–18^, in this study, the utilization of zero-shot CoT prompting resulted in instances where the steps to arrive at an answer were correctly outlined, but the final conclusion based on these steps was inaccurate for certain neurophysiology questions. Therefore, it seems that in the field of neurophysiology, one of the main weaknesses of the LLMs lies in their reasoning capabilities. Further training focused on constructing causal models of physiology could address this issue more effectively than relying solely on statistical associations.

The results of Mahowald et al.^23^ and Tuckute et al.^24^ align with the results we found in our study, indicating that LLMs excel in formal language abilities but exhibit limitations in real-world language understanding and cognitive skills. The Models lack reasoning skills, world knowledge, situation modeling, and social cognition^23,24^. Moreover, Schubert et al. concluded that higher-order cognitive tasks posed significant challenging for both GPT-4 and GPT-3.5^25^. While some researchers express cautious optimism in these cases and express their opinions such as Puchert et al., LLMs have transformed natural language processing and their impressive capabilities, concerns are raised regarding their tendency to generate hallucinations, providing inaccurate information in their responses.

It is emphasized that rigorous evaluation methods are essential to ensure accurate assessment of LLM performance. Evaluations of LLM performance in specific knowledge domains, based on question-and-answer datasets, often rely on a single accuracy metric for the entire field, which hampers transparency and model enhancement^26^. Loconte et al. claimed that while ChatGPT was well known to exhibit outstanding performance in generative linguistic tasks, its performance on prefrontal tests exhibited variability, as they reached the results, with some tests yielding results well above average, others falling in the lower range, and some showing significant impairment^27^. These diverse perspectives underscore the need for a nuanced understanding of LLMs capabilities and limitations across different cognitive tasks and domains.

Overall, the study findings demonstrate that LLMs like ChatGPT, Bard, and Claude have achieved impressive proficiency in responding neurophysiology questions, however, they still face challenges in some aspects of knowledge application, reasoning, and integration. It is evident that there is room for improvement in how these models operate, particularly in answering complex and ambiguous questions that require multistep reasoning and integration of knowledge across diverse topics. The variability observed among different models also highlights the need for ongoing evaluation. As LLMs continue to evolve, rigorous assessment across various knowledge domains will be essential for their continued enhancement and effectiveness.

## Conclusion

This study provides insights into the capabilities of LLMs in answering neurophysiology questions. The results indicate that ChatGPT, Bard, and Claude can successfully address numerous fundamental concepts but face challenges when it comes to more complex reasoning and integration and synthesizing information of knowledge across different topics.

Overall, the models demonstrated relatively strong performance on general neurophysiology and motor system questions with moderate proficiency in sensory neurophysiology. However, they struggled with integrative questions requiring multistep inference. There was no significant difference between languages or cognitive levels. Nevertheless, qualitative analysis revealed inconsistencies and deficiencies, indicating that the models rely heavily on memorization rather than a profound conceptual grasp.

The incorrect responses underscore shortcomings in reasoning, discerning key information, considering the level of importance and priority levels, lack of sufficient information specially in Persian and handling unfamiliar questions. Tailored training focusing on causal physiologic models instead of statistical associations and utilizing reliable sources in various languages could help overcome these limitations. As LLMs advance, rigorous multidisciplinary assessments will be essential to gauge progress and measure advancements.

This study provides a robust evaluation methodology and benchmark for future research aimed at enhancing the neurophysiology knowledge and reasoning competence of these models. The insights can inform efforts to refine LLMs through advanced training techniques and the evaluation of complex integrative tasks. By focusing on targeted improvements, these models hold immense promise in advancing neurophysiology education, research, and clinical practice. The study's findings pave the way for further advancements in LLM technology, ultimately benefiting the field of neurophysiology and beyond.

### Supplementary Information

Supplementary Tables.

Supplementary Information 2.

## Data Availability

The authors declare that there is no relevant data available for this study. All data used in the analysis and preparation of this manuscript have been included in the manuscript.
