# Supplementary material for: Evaluating the strengths and weaknesses of large language models in answering neurophysiology questions
Source: Sci Rep. 2024 May 11;14:10785. doi: 10.1038/s41598-024-60405-y (PMC11088627; doi:10.1038/s41598-024-60405-y)
Supplement: Supplementary file 1 — Supplementary Tables. [file 41598_2024_60405_MOESM1_ESM.docx]

Supplementary 1

Table 1- Questions in the field of neurophysiology and their categorization based on topics and cognitive skills

| No. | Cognitive skill | Question |
| --- | --- | --- |
| General_1 | Lower-order | What is the most important nucleus originating from the adrenergic CNS pathway? |
| General_2 | Lower-order | If there are three neurons A, B, and C connected such that neuron A connects to B and neuron B connects to C, and if neuron A is glutamatergic, neuron B is GABAergic, and neuron C is enkephalinergic. When neuron A is stimulated, will the signal transmitted from neuron B to neuron C be EPSP or IPSP? |
| General_3 | Lower-order | Stimulation of which adrenergic receptor mainly has a regulatory role in presynaptic release?  A) Alpha 1  B) Alpha 2  C) Beta 1  D) Beta 2 |
| General_4 | Higher-order | Is the LTP mechanism mostly mediated by glutamatergic pathways? |
| General_5 | Higher-order | Is myelination of postganglionic sympathetic fibers done by Schwann cells? |
| Sensory_1 | Lower-order | Is the sensation of cold, like acute pain and unlike heat sensation, transmitted through myelinated Aδ (A delta) fibers? |
| Sensory_2 | Lower-order | Are sexual sensations mostly transmitted through the posterior column - medial lemniscus? |
| Sensory_3 | Lower-order | State key components, including nuclei and neurotransmitters, in the central nervous system analgesic pathway? |
| Sensory_4 | Higher-order | Which sensation is NOT transmitted through anterolateral pathway?  A) Chronic pain  B) Cold sensation C) Touch sensation from Meissner receptor  D) Touch sensation from Ruffini receptors |
| Sensory_5 | Higher-order | Cortical areas related to sensations transmitted through which sensory pathways have a more precise and extensive topographical map?  A) Posterior column - medial lemniscus pathway  B) Paleospinothalamic pathway C) Anterior pathway  D) Lateral pathway |
| Motor_1 | Lower-order | Can pontine reticular nucleus receive excitatory signals from the cerebellar fastigial nucleus? |
| Motor_2 | Lower-order | Compared to the vermis, does the cerebellar intermedial area have a greater role in movement precision? |
| Motor _3 | Lower-order | Is hypersexuality seen in Kluver-Bucy syndrome? |
| Motor_4 | Higher-order | Does microinjection of glutamate into the medullary reticular nucleus cause relaxation of axial muscles? |
| Motor_5 | Higher-order | Does stimulation of paraventricular nuclei produce effects similar to amygdala stimulation? |
| Integrative_1 | Lower-order | In medical science and neurophysiology, is knowing "my birthday is January 10, 1998" an example of semantic explicit memory? |
| Integrative _2 | Lower-order | In dementia, neurons secreting which neurotransmitters are more destroyed? |
| Integrative _3 | Lower-order | Do X cells have the highest abundance among retinal ganglion cells? |
| Integrative _4 | Higher-order | In medical science and neurophysiology, which of the following represents explicit memory?  A) The Shahnameh is the masterpiece of the great Iranian poet named Ferdowsi B) Today I arrived about 7 minutes late to physiology class. I'm usually late for classes.  C) In 2010 my house had a major fire  D) One of my elementary school friends' last name ended in "Abadi" or "Abadian" |
| Integrative _5 | Higher-order | Deficiency of which amino acid can cause sleep disruption?  A) Trp  B) Leu  C) Ala  D) Gly |

Table 2- Questions and their scores

| **Language** | **LLM** | **Rater** | **General_1** | **General_2** | **General_3** | **General_4** | **General_5** | **Sensory_1** | **Sensory_2** | **Sensory_3** | **Sensory_4** | **Sensory_5** | **Motor_1** | **Motor_2** | **Motor_3** | **Motor_4** | **Motor_5** | **Integrative_1** | **Integrative _2** | **Integrative _3** | **Integrative _4** | **Integrative _5** |
| --- | --- | --- | --- | --- | --- | --- | --- | --- | --- | --- | --- | --- | --- | --- | --- | --- | --- | --- | --- | --- | --- | --- |
| English | Bard | Rater1 | 5 | 5 | 5 | 5 | 5 | 5 | 5 | 4 | 5 | 5 | 5 | 5 | 5 | 3.5 | 5 | 0 | 4 | 4.5 | 4.5 | 4 |
|  |  | Rater2 | 5 | 5 | 5 | 5 | 4 | 5 | 5 | 3 | 5 | 5 | 5 | 5 | 5 | 4 | 5 | 1 | 5 | 5 | 5 | 5 |
|  |  | Rater3 | 5 | 5 | 5 | 5 | 4.5 | 5 | 5 | 3 | 5 | 5 | 5 | 5 | 5 | 5 | 5 | 1 | 4 | 4 | 4 | 3 |
|  | ChatGPT | Rater1 | 5 | 5 | 5 | 5 | 1 | 5 | 3 | 5 | 5 | 5 | 5 | 5 | 5 | 0 | 4 | 2 | 3.5 | 2 | 1.5 | 5 |
|  |  | Rater2 | 5 | 5 | 5 | 5 | 1 | 5 | 4 | 4 | 5 | 5 | 5 | 5 | 5 | 1 | 5 | 3 | 4 | 3 | 2 | 5 |
|  |  | Rater3 | 5 | 5 | 5 | 5 | 0 | 4 | 3 | 4 | 5 | 5 | 5 | 5 | 5 | 0 | 3 | 2 | 2.5 | 1 | 1 | 5 |
|  | Claude | Rater1 | 5 | 5 | 5 | 5 | 5 | 4 | 5 | 3.5 | 5 | 5 | 4 | 5 | 4 | 0 | 3 | 5 | 3.5 | 5 | 2 | 5 |
|  |  | Rater2 | 5 | 5 | 5 | 5 | 5 | 5 | 5 | 4 | 5 | 5 | 4 | 5 | 4.5 | 0 | 4 | 5 | 5 | 5 | 2 | 5 |
|  |  | Rater3 | 5 | 5 | 5 | 5 | 4 | 4 | 5 | 2 | 5 | 5 | 5 | 5 | 3 | 0 | 3 | 5 | 4 | 5 | 1 | 5 |
| Average | | | 5.0 | 5.0 | 5.0 | 5.0 | 3.3 | 4.7 | 4.4 | 3.6 | 5.0 | 5.0 | 4.8 | 5.0 | 4.6 | 1.5 | 4.1 | 2.7 | 3.9 | 3.8 | 2.6 | 4.7 |
| Persian | Bard | Rater1 | 5 | 5 | 5 | 5 | 0 | 0 | 0 | 1 | 0 | 5 | 4 | 5 | 5 | 5 | 5 | 1 | 3 | 5 | 3 | 5 |
|  |  | Rater2 | 5 | 5 | 5 | 5 | 0 | 0 | 0 | 2 | 0 | 5 | 4 | 5 | 5 | 5 | 4 | 0 | 4 | 5 | 4 | 5 |
|  |  | Rater3 | 5 | 5 | 5 | 5 | 0 | 0 | 0 | 0 | 0 | 5 | 5 | 5 | 5 | 5 | 5 | 0 | 4 | 5 | 2 | 5 |
|  | ChatGPT | Rater1 | 5 | 5 | 5 | 5 | 0 | 5 | 0 | 3.5 | 0 | 5 | 5 | 4 | 5 | 5 | 5 | 1 | 3 | 3 | 3 | 5 |
|  |  | Rater2 | 5 | 5 | 5 | 5 | 0 | 5 | 0 | 4 | 1 | 5 | 5 | 5 | 5 | 5 | 5 | 0 | 4 | 4 | 3 | 5 |
|  |  | Rater3 | 5 | 5 | 5 | 5 | 0 | 5 | 0 | 2 | 0 | 5 | 5 | 4 | 5 | 5 | 5 | 0 | 3 | 3 | 2 | 5 |
|  | Claude | Rater1 | 5 | 5 | 5 | 5 | 1 | 5 | 0 | 4 | 0 | 5 | 5 | 5 | 5 | 5 | 5 | 1 | 3.5 | 4 | 3 | 5 |
|  |  | Rater2 | 5 | 5 | 5 | 5 | 2 | 4 | 0 | 4 | 0 | 5 | 4 | 5 | 5 | 4 | 4 | 0 | 4 | 4 | 4 | 5 |
|  |  | Rater3 | 5 | 5 | 5 | 5 | 0 | 5 | 0 | 3 | 0 | 5 | 5 | 5 | 5 | 5 | 5 | 0 | 3 | 3 | 3 | 5 |
| Average | | | 5.0 | 5.0 | 5.0 | 5.0 | 1.8 | 3.9 | 2.2 | 3.1 | 2.6 | 5.0 | 4.7 | 4.9 | 4.8 | 3.2 | 4.4 | 1.5 | 3.7 | 3.9 | 2.8 | 4.8 |
