# Supplementary material for: Evaluating the strengths and weaknesses of large language models in answering neurophysiology questions
Source: Sci Rep. 2024 May 11;14:10785. doi: 10.1038/s41598-024-60405-y (PMC11088627; doi:10.1038/s41598-024-60405-y)
Supplement: Supplementary file 2 — Supplementary Information 2. [file 41598_2024_60405_MOESM2_ESM.docx]

Supplementary 2 - The English questions along with the answers of the LLMs to these questions

**ChatGPT**

**1-** **Imagine you are an expert physiologist with a specialization in neurophysiology. Answer the following question. {What is the most important nucleus originating from the adrenergic CNS pathway?}. Explain the steps and reasons that lead you to the answer. write your final answer. let's think step by step.**

Certainly! Let's break down the question step by step.

1. **Understanding the Adrenergic CNS Pathway:** The adrenergic central nervous system (CNS) pathway involves the release of neurotransmitters, particularly norepinephrine, from specific nuclei in the brain. These nuclei play a crucial role in regulating various physiological functions, including the "fight or flight" response.
2. **Identification of Key Nuclei:** In the adrenergic CNS pathway, one of the most important nuclei is the **Locus Coeruleus (LC).** The LC is located in the pons of the brainstem and is the principal site for norepinephrine synthesis in the brain.
3. **Reasons for Significance:** The Locus Coeruleus is involved in the modulation of arousal, attention, and stress responses. It projects widely to various brain regions, including the spinal cord, cerebellum, thalamus, and cortex. The release of norepinephrine from the LC has profound effects on the overall state of the nervous system, influencing both cognitive and physiological functions.
4. **Steps in the Adrenergic CNS Pathway:**
   - **Synthesis:** Norepinephrine is synthesized within the neurons of the Locus Coeruleus.
   - **Release:** Upon stimulation, norepinephrine is released into the synaptic cleft.
   - **Receptor Activation:** Norepinephrine binds to adrenergic receptors (mainly α and β receptors) on target cells, transmitting signals.
   - **Effects:** The activation of adrenergic receptors initiates a cascade of physiological responses, including increased heart rate, dilation of airways, and redirection of blood flow to vital organs.
5. **Final Answer:** The most important nucleus originating from the adrenergic CNS pathway is the **Locus Coeruleus (LC).**

In summary, the Locus Coeruleus stands out as a critical nucleus in the adrenergic CNS pathway due to its central role in synthesizing and releasing norepinephrine, which has widespread effects on various physiological and cognitive functions.

**2-** **Imagine you are an expert physiologist with a specialization in neurophysiology. Answer the following question. {If there are three neurons A, B, and C connected such that neuron A connects to B and neuron B connects to C, and if neuron A is glutamatergic, neuron B is GABAergic, and neuron C is enkephalinergic. When neuron A is stimulated, will the signal transmitted from neuron B to neuron C be EPSP or IPSP?}. Explain the steps and reasons that lead you to the answer. write your final answer. let's think step by step.**

Certainly, let's break down the steps to understand the flow of signals in this neuronal circuit:

1. **Neuron A (glutamatergic):** Glutamate is an excitatory neurotransmitter. When neuron A is stimulated, it releases glutamate into the synapse.
2. **Neuron B (GABAergic):** GABA (gamma-aminobutyric acid) is an inhibitory neurotransmitter. Neuron B, being GABAergic, inhibits its target neurons. When neuron B receives the excitatory signal (glutamate) from neuron A, it releases GABA into the synapse.
3. **Neuron C (enkephalinergic):** Enkephalin is an endogenous opioid that can modulate pain perception and neurotransmission. Neuron C, being enkephalinergic, is influenced by the neurotransmitter released by neuron B.

Now, let's deduce the effect of neuron A's stimulation on the signal transmitted from neuron B to neuron C:

- **Neuron A (glutamatergic) → Neuron B (GABAergic):** Neuron A excites neuron B by releasing glutamate.
- **Neuron B (GABAergic) → Neuron C (enkephalinergic):** Neuron B inhibits neuron C by releasing GABA.

Therefore, the overall effect is inhibitory. The signal transmitted from neuron B to neuron C is an Inhibitory Postsynaptic Potential (IPSP).

**Final Answer: The signal transmitted from neuron B to neuron C will be an IPSP.**

**3-** **Imagine you are an expert physiologist with a specialization in neurophysiology. Answer the following question. {Stimulation of which adrenergic receptor mainly has a regulatory role in presynaptic release? A) Alpha 1 B) Alpha 2 C) Beta 1 D) Beta 2 }. Explain the steps and reasons that lead you to the answer. write your final answer. let's think step by step.**

Certainly, let's break down the question step by step.

1. **Understand Adrenergic Receptors:** Adrenergic receptors are part of the sympathetic nervous system and respond to the neurotransmitter adrenaline (epinephrine) and norepinephrine. These receptors are categorized into three main types: Alpha 1, Alpha 2, and Beta receptors (Beta 1 and Beta 2).
2. **Presynaptic Regulation:** Presynaptic regulation refers to the control of neurotransmitter release from the neuron before it reaches the synapse. In this context, the question is asking about the adrenergic receptor that mainly regulates presynaptic release.
3. **Review Receptor Functions:**
   - **Alpha 1 receptors:** Mainly postsynaptic, involved in smooth muscle contraction.
   - **Alpha 2 receptors:** Located presynaptically, usually inhibiting the release of neurotransmitters, including norepinephrine.
   - **Beta 1 receptors:** Primarily found in the heart, stimulating the heart rate and force of contraction.
   - **Beta 2 receptors:** Mainly found in the lungs and blood vessels, causing bronchodilation and vasodilation.
4. **Identify the Receptor with Presynaptic Regulation:** The adrenergic receptor primarily involved in presynaptic inhibition (regulating the release of neurotransmitters) is Alpha 2.
5. **Final Answer:** Therefore, the correct answer to the question is: **B) Alpha 2.**

Alpha 2 receptors, when activated, inhibit further release of norepinephrine, creating a negative feedback loop that helps modulate the sympathetic nervous system.

**4- Imagine you are an expert physiologist with a specialization in neurophysiology. Answer the following question. {Is the LTP mechanism mostly mediated by glutamatergic pathways?}. Explain the steps and reasons that lead you to the answer. write your final answer. let's think step by step.**

Certainly, let's break down the question step by step.

1. **Understanding LTP (Long-Term Potentiation):**
   - LTP is a persistent increase in synaptic strength that results from high-frequency stimulation of a neuron's input.
   - It is a key mechanism in synaptic plasticity, believed to underlie learning and memory.
2. **Glutamatergic Pathways and LTP:**
   - Glutamate is the primary excitatory neurotransmitter in the central nervous system.
   - Most excitatory synapses in the mammalian brain use glutamate, and it acts on ionotropic receptors such as NMDA and AMPA receptors.
3. **NMDA Receptor Activation:**
   - During high-frequency stimulation, NMDA receptors are particularly crucial.
   - NMDA receptors are both ligand-gated and voltage-dependent, allowing them to detect coincident pre- and postsynaptic activity.
   - Calcium influx through activated NMDA receptors is a key trigger for LTP.
4. **AMPA Receptor Trafficking:**
   - AMPA receptors also play a significant role in LTP.
   - Calcium influx through NMDA receptors leads to changes in AMPA receptor trafficking, contributing to the increase in synaptic strength.
5. **Synaptic Tagging and Protein Synthesis:**
   - LTP involves the activation of various intracellular signaling pathways.
   - The "synaptic tagging" hypothesis suggests that synapses are "tagged" during LTP induction, making them capable of capturing newly synthesized proteins necessary for the maintenance of potentiation.
6. **Neurotransmitter Specificity:**
   - While other neurotransmitters can modulate synaptic transmission, the majority of evidence supports glutamatergic pathways as the primary mediators of LTP.
7. **Final Answer:**
   - Yes, the LTP mechanism is mostly mediated by glutamatergic pathways.
   - Glutamate, acting on NMDA and AMPA receptors, plays a central

**5- Imagine you are an expert physiologist with a specialization in neurophysiology. Answer the following question. {Is myelination of postganglionic sympathetic fibers done by Schwann cells?}. Explain the steps and reasons that lead you to the answer. write your final answer. let's think step by step.**

Certainly, let's break down the question step by step.

1. Identification of the Nervous System Component:
   - The question refers to postganglionic sympathetic fibers, which are part of the autonomic nervous system.
2. Understanding Myelination:
   - Myelination is the process of forming a myelin sheath around nerve fibers, which helps in the transmission of nerve impulses.
3. Identification of Cells Responsible for Myelination:
   - In the peripheral nervous system (PNS), myelination is primarily done by Schwann cells.
4. Location of Myelination:
   - Schwann cells myelinate axons in the peripheral nerves, including those of the autonomic nervous system.
5. Distinguishing between Preganglionic and Postganglionic Fibers:
   - Preganglionic fibers, which extend from the spinal cord to the ganglia, are myelinated by Schwann cells. However, postganglionic fibers, which extend from the ganglia to the target organs, are not myelinated.
6. Reasoning for Lack of Myelination in Postganglionic Fibers:
   - The absence of myelination in postganglionic fibers is functionally relevant. Unmyelinated fibers allow for slower, more diffuse neurotransmission, which is appropriate for the autonomic nervous system's control over involuntary bodily functions.
7. Summarizing the Steps:
   - Identification of the nervous system component (postganglionic sympathetic fibers).
   - Understanding myelination as a process of forming a myelin sheath around nerve fibers.
   - Recognizing Schwann cells as the primary myelinating cells in the peripheral nervous system.
   - Realizing that myelination by Schwann cells is primarily associated with preganglionic fibers, not postganglionic fibers.
   - Appreciating the functional significance of unmyelinated postganglionic fibers in the autonomic nervous system.

Final Answer: No, myelination of postganglionic sympathetic fibers is not done by Schwann cells. Postganglionic fibers in the autonomic nervous system are typically unmyelinated. Myelination in the peripheral nervous system, including the autonomic nervous system, is primarily associated with Schwann cells, but this process is more relevant to preganglionic fibers. The lack of myelination in postganglionic fibers contributes to the specific characteristics of neurotransmission required for autonomic functions.

**6- Imagine you are an expert physiologist with a specialization in neurophysiology. Answer the following question. {Is the sensation of cold, like acute pain and unlike heat sensation, transmitted through myelinated Aδ (A delta) fibers?}. Explain the steps and reasons that lead you to the answer. write your final answer. let's think step by step.**

Certainly, let's break down the question step by step.

1. **Understanding the Types of Peripheral Nerve Fibers:**
   - Aδ (A delta) fibers and C fibers are two main types of peripheral nerve fibers involved in the transmission of temperature and pain sensations.
   - Aδ fibers are myelinated and generally associated with the transmission of sharp, well-localized pain sensations.
   - C fibers are unmyelinated and typically transmit dull, diffuse pain sensations.
2. **Cold Sensation Transmission:**
   - Cold sensation is transmitted through specific temperature-sensitive receptors known as thermoreceptors.
   - The transmission of cold sensation involves the activation of these thermoreceptors in the skin.
3. **Aδ Fibers and Cold Sensation:**
   - Cold sensation, like acute pain, is indeed transmitted through myelinated Aδ fibers.
   - Aδ fibers are responsible for the rapid transmission of signals, and cold sensation, being a sharp and well-localized stimulus, aligns with the characteristics of Aδ fiber transmission.
4. **Heat Sensation:**
   - Heat sensation, on the other hand, is transmitted through both Aδ and C fibers.
   - Aδ fibers are involved in the initial sharp perception of heat, while C fibers transmit the more sustained, burning-type heat sensations.
5. **Summarizing the Steps:**
   - Aδ fibers are myelinated and associated with sharp, well-localized sensations.
   - Cold sensation, similar to acute pain, is sharp and well-localized.
   - Therefore, the sensation of cold is transmitted through myelinated Aδ fibers.
6. **Final Answer:**
   - Yes, the sensation of cold, like acute pain and unlike heat sensation, is transmitted through myelinated Aδ (A delta) fibers.

In conclusion, the specific characteristics of cold sensation align with the properties of Aδ fibers, supporting the notion that Aδ fibers are involved in the transmission of the sensation of cold.

**7- Imagine you are an expert physiologist with a specialization in neurophysiology. Answer the following question. {Are sexual sensations mostly transmitted through the posterior column - medial lemniscus?}. Explain the steps and reasons that lead you to the answer. write your final answer. let's think step by step.**

Certainly! Let's break down the question step by step.

1. **Understanding the Neural Pathways:**
   - The posterior column-medial lemniscus pathway primarily transmits tactile and proprioceptive information from the body to the brain.
   - Sexual sensations involve a complex interplay of sensory, psychological, and hormonal factors.
2. **Involvement of Multiple Pathways:**
   - Sexual sensations are not solely reliant on touch and proprioception; they involve a combination of sensory inputs, including tactile, thermal, and nociceptive signals.
   - The spinothalamic pathway is another major pathway responsible for transmitting temperature and pain sensations, which are also relevant to sexual experiences.
3. **Involvement of Higher Brain Centers:**
   - Sexual sensations also heavily involve higher brain centers, such as the limbic system, which is responsible for emotions and motivation.
   - The limbic system integrates various sensory inputs and plays a crucial role in the emotional and psychological aspects of sexual experiences.
4. **Autonomic Nervous System Involvement:**
   - Sexual responses also engage the autonomic nervous system, which controls involuntary bodily functions. This includes the parasympathetic and sympathetic divisions, influencing aspects like arousal and genital blood flow.
5. **Hormonal Influences:**
   - Hormones, especially sex hormones like testosterone and estrogen, play a pivotal role in sexual sensations. Hormonal signals are not transmitted through the posterior column-medial lemniscus pathway but rather through the bloodstream.

Considering these points, it's evident that sexual sensations involve a complex interplay of sensory, emotional, psychological, and hormonal factors that extend beyond the transmission pathways of the posterior column-medial lemniscus. While touch and proprioception contribute to sexual experiences, they are only part of the broader neural and physiological processes involved.

**Final Answer:** Sexual sensations are not mostly transmitted through the posterior column-medial lemniscus pathway. Instead, they involve a complex integration of sensory inputs, emotional and psychological factors, as well as hormonal influences. The posterior column-medial lemniscus pathway primarily transmits tactile and proprioceptive information, but sexual sensations engage a wider network of neural pathways and physiological processes.

**8- Imagine you are an expert physiologist with a specialization in neurophysiology. Answer the following question. {State key components, including nuclei and neurotransmitters, in the central nervous system analgesic pathway}. Explain the steps and reasons that lead you to the answer. write your final answer. let's think step by step.**

Certainly, let's break down the question and address it step by step.

1. **Understanding the Question:** The question is asking for key components, including nuclei and neurotransmitters, in the central nervous system analgesic pathway. This implies a focus on the neural pathway involved in pain modulation and control.
2. **Identification of Key Components:** In the context of analgesia (pain relief), the central nervous system (CNS) pathway involves several key components. These include specific nuclei within the brain and spinal cord, as well as the neurotransmitters associated with pain modulation.
   - **Periaqueductal Gray (PAG):** The PAG is a key nucleus located in the midbrain. It plays a crucial role in the descending modulation of pain.
   - **Raphe Nuclei:** These nuclei, located in the brainstem, are involved in the release of serotonin, a neurotransmitter associated with pain inhibition.
   - **Substantia Gelatinosa (SG) of the Spinal Cord:** This region of the spinal cord is important for the modulation of pain signals as they ascend towards the brain.
   - **Endorphins and Enkephalins:** These are endogenous opioid neurotransmitters that act as natural painkillers. They bind to opioid receptors in the CNS, particularly in the PAG and the spinal cord, to modulate pain.
3. **Steps in the Analgesic Pathway:** The analgesic pathway involves a descending control mechanism, where signals are modulated as they travel from the site of injury or pain towards the brain.
   - **Activation of PAG:** The process often begins with the activation of the Periaqueductal Gray in response to pain stimuli.
   - **Release of Serotonin:** The Raphe Nuclei release serotonin, which inhibits the transmission of pain signals in the spinal cord.
   - **Opioid Release:** Endorphins and enkephalins are released, binding to opioid receptors in the CNS, particularly in the PAG and spinal cord, resulting in pain modulation.
4. **Reasoning:** The selection of these components is based on the current understanding of the neurophysiology of pain modulation. These structures and neurotransmitters have been extensively studied and are known to play crucial roles in the analgesic pathway.
5. **Final Answer:** In the central nervous system analgesic pathway, key components include the Periaqueductal Gray (PAG), Raphe Nuclei, and the Substantia Gelatinosa (SG) of the spinal cord. Neurotransmitters such as serotonin from the Raphe Nuclei and endorphins/enkephalins act in concert to modulate pain signals, with opioid receptors in the CNS playing a pivotal role in this process.

**9- Imagine you are an expert physiologist with a specialization in neurophysiology. Answer the following question. {Which sensation is not transmitted through the anterolateral pathway? A) Chronic pain B) Cold sensation C) Touch sensation from Meissner receptor D) Touch sensation from Ruffini receptors}. Explain the steps and reasons that lead you to the answer. write your final answer. let's think step by step.**

Certainly! Let's break down the information step by step.

The anterolateral pathway, also known as the spinothalamic tract, is responsible for transmitting pain and temperature sensations. This pathway consists of two main components: the neospinothalamic tract, which transmits fast pain (like a sharp, acute pain), and the paleospinothalamic tract, which transmits slow pain (like a dull, chronic pain) and temperature sensations.

Now, let's analyze the given options:

A) Chronic pain: Chronic pain is associated with slow pain fibers and is transmitted through the paleospinothalamic tract, which is part of the anterolateral pathway. So, chronic pain is transmitted through the anterolateral pathway.

B) Cold sensation: Cold sensation is also transmitted through the paleospinothalamic tract of the anterolateral pathway. So, cold sensation is transmitted through this pathway.

C) Touch sensation from Meissner receptor: Touch sensation from Meissner receptors is not transmitted through the anterolateral pathway. Meissner receptors are part of the dorsal column-medial lemniscal pathway, which is a separate pathway responsible for fine touch and proprioception.

D) Touch sensation from Ruffini receptors: Ruffini receptors are also not associated with the anterolateral pathway. They are part of the dorsal column-medial lemniscal pathway, similar to Meissner receptors.

Therefore, the sensation that is not transmitted through the anterolateral pathway is:

**Final Answer: C) Touch sensation from Meissner receptor**

**10- Imagine you are an expert physiologist with a specialization in neurophysiology. Answer the following question. {Cortical areas related to sensations transmitted through which sensory pathways have a more precise and extensive topographical map? A) Posterior column - medial lemniscus pathway B) Paleospinothalamic pathway C) Anterior pathway D) Lateral pathway }. Explain the steps and reasons that lead you to the answer. write your final answer. let's think step by step.**

Certainly, let's break down the question step by step.

1. **Understand the Sensory Pathways:**
   - The question refers to cortical areas related to sensations, so we're primarily looking at sensory pathways.
   - The options provided are:
     - A) Posterior column - medial lemniscus pathway
     - B) Paleospinothalamic pathway
     - C) Anterior pathway
     - D) Lateral pathway
2. **Consider Topographical Map:**
   - The topographical map in the context of the nervous system refers to the spatial arrangement of sensory neurons in the brain.
   - A more precise and extensive topographical map implies a finer representation of different sensory inputs.
3. **Evaluate Cortical Areas:**
   - Different cortical areas process sensory information, and some are known for having more detailed topographical maps.
4. **Analysis of Pathways:**
   - The Posterior column - medial lemniscus pathway (Option A) is associated with proprioception, vibration, and fine touch. This pathway generally has a well-organized representation in the primary somatosensory cortex, suggesting a detailed topographical map.
   - The Paleospinothalamic pathway (Option B) is associated with poorly localized, aching pain. It may not provide as precise a topographical map.
   - The Anterior pathway (Option C) is not a standard term in neurophysiology, and it might be a distractor.
   - The Lateral pathway (Option D) is not specific, and without further details, it's challenging to determine its relevance.
5. **Consider Precision and Extensiveness:**
   - The pathway that transmits sensations requiring a more precise and extensive topographical map is likely to be one associated with detailed sensory discrimination, such as touch and proprioception.
6. **Final Answer:**
   - Based on the analysis, the pathway most likely associated with a more precise and extensive topographical map for sensations is the **Posterior column - medial lemniscus pathway (Option A).**

In summary, the Posterior column - medial lemniscus pathway is known for transmitting sensory information related to proprioception and fine touch, and its representation in the primary somatosensory cortex suggests a more detailed topographical map. Therefore, the final answer is **A) Posterior column - medial lemniscus pathway.**

**11- Imagine you are an expert physiologist with a specialization in neurophysiology. Answer the following question. {Can pontine reticular nucleus receive excitatory signals from the cerebellar fastigial nucleus?}. Explain the steps and reasons that lead you to the answer. write your final answer. let's think step by step.**

Certainly, let's break down the question step by step:

**Step 1: Understand the Neural Pathway** The pontine reticular nucleus (PRN) and the cerebellar fastigial nucleus (FN) are both components of the central nervous system. To determine whether PRN can receive excitatory signals from the cerebellar fastigial nucleus, we need to understand the neural connections between these structures.

**Step 2: Identify Neural Connections** The cerebellar fastigial nucleus primarily projects its fibers to the brainstem, particularly the vestibular nuclei and the reticular formation. The pontine reticular nucleus is part of the reticular formation in the brainstem.

**Step 3: Consult Existing Knowledge** Referring to existing neurophysiological knowledge, it's known that the cerebellum, including the fastigial nucleus, plays a crucial role in motor control and coordination. The cerebellum communicates with various brainstem nuclei, and the reticular formation is one of them.

**Step 4: Evaluate the Type of Signals** Determine the nature of signals between the cerebellar fastigial nucleus and the pontine reticular nucleus. Excitatory signals typically involve the release of neurotransmitters that increase the likelihood of an action potential in the receiving neuron.

**Step 5: Synthesis of Information** Given that the cerebellum, including the fastigial nucleus, is involved in motor coordination and the pontine reticular nucleus is part of the reticular formation involved in motor control, it is plausible that excitatory signals from the fastigial nucleus could influence the activity of the pontine reticular nucleus.

**Final Answer:** Yes, the pontine reticular nucleus can receive excitatory signals from the cerebellar fastigial nucleus. The anatomical connections and the functional roles of these structures in motor coordination support the possibility of excitatory communication between them.

**12- Imagine you are an expert physiologist with a specialization in neurophysiology. Answer the following question. {Compared to the vermis, does the cerebellar intermedial area have a greater role in movement precision?}. Explain the steps and reasons that lead you to the answer. write your final answer. let's think step by step.**

Certainly! Let's break down the question step by step.

1. **Understand the Brain Anatomy:** First, we need to understand the basic anatomy of the cerebellum. The cerebellum is divided into three parts: the lateral hemisphere, the vermis (which is the midline portion), and the flocculonodular lobe. The cerebellar interposed nucleus or interpositus nucleus is located within the cerebellar intermedial area.
2. **Function of the Vermis:** The vermis is traditionally associated with controlling the axial musculature and plays a crucial role in maintaining posture and coordinating gross movements.
3. **Function of the Cerebellar Intermedial Area:** The cerebellar intermedial area, particularly the interpositus nucleus, is more involved in the control of limb movements and is linked to the precision of distal movements, such as those of the fingers and hands.
4. **Comparing Roles:** To determine which area has a greater role in movement precision, we need to consider the nature of the movements. If we are discussing precise, fine motor movements, such as those involved in skilled tasks like playing a musical instrument or typing, the cerebellar intermedial area is likely to play a more significant role due to its association with distal limb control.
5. **Clinical Observations:** Observations from clinical studies and cases where damage to either the vermis or the cerebellar intermedial area has occurred can provide valuable insights. Lesions in the cerebellar intermedial area often result in issues related to fine motor control.
6. **Neurophysiological Evidence:** Neurophysiological studies involving recording neuronal activity in these cerebellar regions during different motor tasks can also provide evidence of their specific roles in movement control and precision.
7. **Synthesis of Information:** Based on the anatomical location, known functions, clinical observations, and neurophysiological evidence, it can be concluded that the cerebellar intermedial area, particularly the interpositus nucleus, has a greater role in movement precision compared to the vermis.
8. **Final Answer:** Yes, compared to the vermis, the cerebellar intermedial area, especially the interpositus nucleus, has a greater role in movement precision. This conclusion is drawn from the specialization of the intermedial area in distal limb control and the observed consequences of lesions or dysfunction in this region in fine motor tasks.

**13- Imagine you are an expert physiologist with a specialization in neurophysiology. Answer the following question. {Is hypersexuality seen in Kluver-Bucy syndrome?}. Explain the steps and reasons that lead you to the answer. write your final answer. let's think step by step.**

Certainly, let's break down the question and arrive at a comprehensive answer.

**Step 1: Understand Kluver-Bucy Syndrome**

Kluver-Bucy syndrome is a rare neurobehavioral disorder associated with bilateral lesions in the medial temporal lobe, particularly the amygdala. It was first observed in monkeys and later in humans. The syndrome is characterized by a range of behavioral changes, including hypersexuality.

**Step 2: Linking Hypersexuality to Neurological Changes**

The amygdala is a crucial part of the brain involved in emotional processing and regulation, including sexual behavior. Bilateral lesions in the amygdala, as seen in Kluver-Bucy syndrome, can lead to disinhibited behaviors, including hypersexuality. The loss of inhibitions may result in increased sexual activity and reduced recognition of appropriate social contexts for such behaviors.

**Step 3: Reviewing Clinical Observations**

Clinical observations and case studies of individuals with Kluver-Bucy syndrome often report hypersexual behaviors among other symptoms such as visual agnosia, oral exploration of objects, and changes in dietary habits. These observations further support the association between Kluver-Bucy syndrome and hypersexuality.

**Final Answer:**

Yes, hypersexuality is commonly seen in Kluver-Bucy syndrome. The bilateral lesions in the amygdala, a key brain region for emotional regulation, lead to disinhibition and inappropriate expression of sexual behaviors. Clinical evidence and case studies consistently support the presence of hypersexuality as one of the behavioral manifestations of Kluver-Bucy syndrome.

**14-** **Imagine you are an expert physiologist with a specialization in neurophysiology. Answer the following question. {Does microinjection of glutamate into the medullary reticular nucleus cause relaxation of axial muscles?}. Explain the steps and reasons that lead you to the answer. write your final answer. let's think step by step.**

Certainly! Let's break down the question step by step and consider the relevant physiological processes involved.

1. **Understand the Basics:** First, we need to understand the role of the medullary reticular nucleus in the central nervous system. The medullary reticular nucleus is involved in the regulation of various autonomic functions, including those related to respiration and cardiovascular control.
2. **Role of Glutamate:** Glutamate is an excitatory neurotransmitter in the central nervous system. Microinjection of glutamate typically leads to the depolarization of neurons, causing an increase in neural activity.
3. **Axial Muscle Function:** Axial muscles are responsible for maintaining posture and controlling movements of the trunk. The state of muscle contraction (relaxation or contraction) is primarily regulated by the balance of excitatory and inhibitory signals from the nervous system.
4. **Connections to Axial Muscles:** To determine the effect of microinjection of glutamate into the medullary reticular nucleus on axial muscles, we need to consider the neural connections between the medullary reticular nucleus and the motor neurons that innervate axial muscles.
5. **Neurotransmitter Release to Axial Muscles:** In a simplified model, if the medullary reticular nucleus is connected to motor neurons that control axial muscles, the release of excitatory neurotransmitters (such as glutamate) could lead to increased neural activity in those motor neurons, potentially causing muscle contraction rather than relaxation.
6. **Consideration of Inhibitory Pathways:** It's also crucial to consider whether there are inhibitory pathways involved. In some cases, activation of certain neural pathways may lead to the release of inhibitory neurotransmitters, causing muscle relaxation.
7. **Experimental Evidence:** To answer the question definitively, experimental evidence is needed. Studies involving microinjection of glutamate into the medullary reticular nucleus, along with electromyographic (EMG) recordings of axial muscle activity, would provide insights into the specific effects on muscle tone.
8. **Conclusion:** In the absence of specific experimental data, based on our understanding of neurophysiology, it's more likely that microinjection of glutamate into the medullary reticular nucleus would lead to increased neural activity and, consequently, muscle contraction rather than relaxation of axial muscles.

**Final Answer:** Based on the current understanding of neurophysiology, it is more likely that microinjection of glutamate into the medullary reticular nucleus would cause an increase in neural activity, potentially leading to the contraction rather than relaxation of axial muscles. However, to confirm this, experimental studies involving glutamate microinjection and monitoring of axial muscle activity would be necessary.

**15- Imagine you are an expert physiologist with a specialization in neurophysiology. Answer the following question. {Does stimulation of paraventricular nuclei produce effects similar to amygdala stimulation?}. Explain the steps and reasons that lead you to the answer. write your final answer. let's think step by step.**

Certainly, let's break down the question and address it step by step:

1. **Understanding the Structures:**
   - The paraventricular nuclei (PVN) and the amygdala are distinct structures within the brain.
   - The PVN is part of the hypothalamus and plays a crucial role in regulating various autonomic functions, including the stress response and the release of hormones like oxytocin and vasopressin.
   - The amygdala, on the other hand, is involved in processing emotions, particularly fear and pleasure responses.
2. **Neurophysiological Effects:**
   - Stimulation of the amygdala is associated with emotional responses, particularly fear responses. It can lead to the activation of the sympathetic nervous system, triggering the "fight or flight" response.
   - Stimulation of the PVN can also influence autonomic functions and hormone release, but its primary role is often linked to the regulation of stress responses and homeostasis.
3. **Connections Between Structures:**
   - The amygdala and the PVN are interconnected, forming a network involved in the regulation of stress responses. The amygdala can signal the PVN to release stress hormones.
4. **Differences in Function:**
   - While both structures are involved in aspects of the stress response, the amygdala is more directly linked to emotional processing, including fear, whereas the PVN has a broader role in regulating various physiological functions.
5. **Experimental Evidence:**
   - Experimental studies involving stimulation of the amygdala and PVN have been conducted using techniques such as electrical stimulation or optogenetics.
   - These studies reveal distinct patterns of physiological responses and behavioral outcomes associated with stimulation of each structure.
6. **Conclusion:**
   - Stimulation of the paraventricular nuclei may produce effects related to stress and autonomic functions, but the nature of these effects is likely to differ from the emotional responses associated with amygdala stimulation.
   - The amygdala is more directly involved in emotional processing, especially fear, whereas the PVN is part of a broader regulatory network for maintaining homeostasis.

**Final Answer:** Stimulation of the paraventricular nuclei may influence stress responses and autonomic functions, but the effects are not expected to be identical to those produced by amygdala stimulation. The amygdala is primarily associated with emotional processing, particularly fear, while the paraventricular nuclei play a role in the broader regulation of physiological functions and the stress response. Experimental evidence supports the distinct functional roles of these two brain regions.

**16- Imagine you are an expert physiologist with a specialization in neurophysiology. Answer the following question. {In medical science and neurophysiology, is knowing "my birthday is January 10, 1998" an example of semantic explicit memory?}. Explain the steps and reasons that lead you to the answer. write your final answer. let's think step by step.**

Certainly, let's break down the question step by step.

1. **Define Semantic Explicit Memory:**
   - Semantic memory refers to the general knowledge and facts that a person has accumulated over their lifetime.
   - Explicit memory involves the conscious, intentional recollection of information.
2. **Analyze the Statement: "My birthday is January 10, 1998":**
   - The statement involves personal information, specifically the birthdate of the individual (January 10, 1998).
3. **Determine if it's Semantic Memory:**
   - The information about the birthdate is personal and specific to the individual.
   - However, this information doesn't fall under the category of general knowledge or facts that apply universally. It is specific to the person.
4. **Consider Explicit Memory:**
   - The statement involves a conscious recollection of a specific piece of personal information, the birthdate.
5. **Conclusion:**
   - The statement "My birthday is January 10, 1998" is an example of explicit memory since it involves the intentional recollection of personal information.
   - However, it may not be considered an example of semantic memory because it is not a general fact or knowledge that applies broadly.

**Final Answer:**

- Knowing "My birthday is January 10, 1998" is an example of explicit memory, but not semantic memory, as it involves the conscious recall of specific personal information rather than general knowledge.

**17- Imagine you are an expert physiologist with a specialization in neurophysiology. Answer the following question. {In dementia, neurons secreting which neurotransmitters are more destroyed}. Explain the steps and reasons that lead you to the answer. write your final answer. let's think step by step.**

Certainly! Let's approach this question step by step.

1. **Understanding Dementia:** Dementia is a broad term for a set of symptoms, including memory loss and cognitive decline, that result from damage to the brain. There are various types of dementia, such as Alzheimer's disease, vascular dementia, and Lewy body dementia.
2. **Neurotransmitters and Neuron Destruction:** Neurotransmitters are chemical messengers that transmit signals between neurons (nerve cells) in the brain. In dementia, neuron damage occurs, leading to a decline in cognitive function. Different types of dementia are associated with specific patterns of neuron damage.
3. **Alzheimer's Disease:** Alzheimer's disease is the most common cause of dementia. In Alzheimer's, there is an accumulation of abnormal proteins, such as beta-amyloid plaques and tau tangles. These affect areas of the brain involved in memory and cognition. The neurotransmitter acetylcholine is significantly depleted in Alzheimer's disease due to damage to cholinergic neurons.
4. **Cholinergic Neurons:** Acetylcholine is a crucial neurotransmitter involved in memory and learning. Cholinergic neurons, which release acetylcholine, are particularly vulnerable in Alzheimer's disease. The destruction of these neurons contributes to the cognitive decline seen in individuals with Alzheimer's.
5. **Final Answer:** In dementia, particularly in Alzheimer's disease, neurons secreting acetylcholine are more destroyed. The specific involvement of cholinergic neurons and the depletion of acetylcholine contribute to the characteristic cognitive decline observed in individuals with Alzheimer's disease.

So, to answer the question:

**In dementia, neurons secreting acetylcholine are more destroyed.**

**18 -Imagine you are an expert physiologist with a specialization in neurophysiology. Answer the following question. {Do X cells have the highest abundance among retinal ganglion cells?}. Explain the steps and reasons that lead you to the answer. write your final answer. let's think step by step.**

Certainly! Let's break down the question and approach it step by step.

1. **Understand the Question:**
   - The question is asking about the abundance of X cells among retinal ganglion cells.
2. **Identify the Types of Retinal Ganglion Cells:**
   - Retinal ganglion cells can be classified into different types based on various criteria such as morphology, function, and response to visual stimuli.
3. **Review Existing Knowledge:**
   - Based on current knowledge, there isn't a specific mention of "X cells" among retinal ganglion cells. However, we know that there are different types, such as M cells (magnocellular), P cells (parvocellular), and other specialized cells with unique functions.
4. **Consider Abundance:**
   - Abundance could refer to the numerical prevalence of a specific type of cell among retinal ganglion cells. This could be influenced by factors like function, importance in visual processing, or evolutionary significance.
5. **Assess Function and Importance:**
   - Consider the function and importance of each type of retinal ganglion cell. For example, some cells might be more involved in processing color information, while others might be crucial for detecting motion.
6. **Evaluate Research Studies:**
   - Examine any recent studies or literature that may provide insights into the prevalence of different retinal ganglion cell types, including X cells if they exist.
7. **Consider Evolutionary Aspects:**
   - Evolutionary significance could play a role in determining the abundance of certain cell types. Cells that are essential for survival or have evolved for specific visual tasks might be more abundant.
8. **Consider Technological Advancements:**
   - Advances in imaging and neurophysiological techniques may have provided more detailed information about the types and abundance of retinal ganglion cells.

**Final Answer:**

- Based on the current state of knowledge up to my last update in January 2022, there isn't a specific mention of "X cells" among retinal ganglion cells. The abundance of retinal ganglion cell types, including any labeled as X cells, would depend on various factors such as function, importance in visual processing, and evolutionary significance. Without specific information about X cells, it is not possible to definitively state whether they have the highest abundance among retinal ganglion cells. Additional research and information on X cells are needed to provide a more accurate answer.

**19- Imagine you are an expert physiologist with a specialization in neurophysiology. Answer the following question. {In medical science and neurophysiology, which of the following represents explicit memory? A) The Shahnameh is the masterpiece of the great Iranian poet named Ferdowsi B) Today I arrived about 7 minutes late to physiology class. C) In 2010 my house had a major fire D) One of my elementary school friends' lastname ended in "Abadi" or "Abadian".} Explain the steps and reasons that lead you to the answer. write your final answer. let's think step by step.**

Certainly, let's break down the question step by step.

Explicit memory refers to the conscious, intentional recollection of information. It is divided into episodic and semantic memory. Episodic memory involves personal experiences, while semantic memory involves general knowledge. Now, let's analyze the given options:

A) "The Shahnameh is the masterpiece of the great Iranian poet named Ferdowsi."

- This statement contains factual information about a literary work and its author. It represents semantic memory, as it is general knowledge.

B) "Today I arrived about 7 minutes late to physiology class."

- This statement is a personal experience, representing episodic memory. It involves the recollection of a specific event from the individual's life.

C) "In 2010, my house had a major fire."

- Similar to option B, this statement is a personal experience, specifically an episodic memory of a significant event in the person's life.

D) "One of my elementary school friends' last name ended in 'Abadi' or 'Abadian'."

- This statement involves the recall of specific information about a friend's last name, representing episodic memory.

Therefore, options B, C, and D involve explicit memory, as they are personal experiences. However, option A involves semantic memory, which is not considered explicit memory.

Final Answer: Options B, C, and D represent explicit memory, but option A does not.

**20- Imagine you are an expert physiologist with a specialization in neurophysiology. Answer the following question. {Deficiency of which amino acid can cause sleep disruption? A) Trp B) Leu C) Ala D) Gly }. Explain the steps and reasons that lead you to the answer. write your final answer. let's think step by step.**

Certainly! Let's analyze each option step by step:

A) Trp (Tryptophan): Tryptophan is a precursor for serotonin and melatonin, which are neurotransmitters involved in sleep regulation. A deficiency in tryptophan could potentially lead to a decrease in the production of these sleep-regulating neurotransmitters.

B) Leu (Leucine): Leucine is an essential amino acid involved in protein synthesis and not directly linked to sleep regulation. A deficiency in leucine is less likely to cause sleep disruption.

C) Ala (Alanine): Alanine is a non-essential amino acid and is not directly involved in the synthesis of neurotransmitters related to sleep. A deficiency in alanine is unlikely to affect sleep.

D) Gly (Glycine): Glycine is another amino acid that plays a role in neurotransmitter function. It acts as an inhibitory neurotransmitter in the central nervous system and has been associated with improved sleep quality. A deficiency in glycine might disrupt this balance.

Considering the options and their potential impact on sleep, the most plausible answer is:

**Final Answer: A) Trp (Tryptophan)**

A deficiency in tryptophan could lead to reduced synthesis of serotonin and melatonin, affecting the sleep-wake cycle.

**Bard**

**1- Imagine you are an expert physiologist with a specialization in neurophysiology. Answer the following question. {What is the most important nucleus originating from the adrenergic CNS pathway?}. Explain the steps and reasons that lead you to the answer. write your final answer. let's think step by step.**

Determining the Most Important Nucleus of the Adrenergic CNS Pathway: A Step-by-Step Approach

As a neurophysiologist, I would approach this question in a methodical way, considering several factors:

Step 1: Define "importance" and the context of the adrenergic CNS pathway.

- Importance: What metric are we using to define "most important"? Is it the broadest influence on downstream targets, the highest concentration of pre-motor neurons, or the most critical role in a specific physiological response?
- Context: Are we focusing on a particular organ system (e.g., cardiovascular, respiratory) or considering the broad spectrum of adrenergic regulation?

Step 2: Review the key nuclei of the adrenergic CNS pathway.

- Medulla oblongata:
  - Nucleus of the solitary tract (NTS): Receives and integrates visceral sensory information for reflex control of cardiovascular, respiratory, and other functions.
  - Dorsal vagal complex (DVC): Contains pre-motor neurons for parasympathetic control of various organs. While not technically adrenergic, the DVC interacts with and modulates adrenergic outflow.
- Brainstem nuclei:
  - A1 and A2 regions: Project to the spinal cord and control preganglionic sympathetic neurons for widespread peripheral effects.
  - Locus coeruleus (LC): Modulates arousal and attention; sends projections to various brain regions influencing cardiovascular and other functions.
- Hypothalamus:
  - Preoptic area (POA): Involved in thermoregulation and cardiovascular control.
  - Paraventricular nucleus (PVN): Major integrative center for autonomic control, including both sympathetic and parasympathetic outflow.

Step 3: Analyze the relative influence and roles of each nucleus.

- NTS: Broadly receives and integrates information, but its outputs are modulated by other nuclei.
- DVC: Important for parasympathetic control, but less directly involved in adrenergic signaling.
- A1/A2 regions: Directly control spinal sympathetic neurons, but lack broader integration and modulation capabilities.
- LC: Important for arousal and some physiological responses, but has a more diffuse influence compared to other nuclei.
- POA: Primarily involved in thermoregulation, with a more specific role compared to other nuclei.
- PVN: Integrates inputs from various systems, coordinates both sympathetic and parasympathetic outflow, and directly projects to preganglionic sympathetic neurons.

Step 4: Conclusion based on defined "importance" and context.

- If "importance" is defined as the broadest influence on downstream targets: then the PVN emerges as the most crucial nucleus, due to its central role in integrating and coordinating autonomic responses.
- If "importance" is defined as the highest concentration of pre-motor neurons: then the A1/A2 regions could be considered, as they directly control spinal sympathetic output.
- If "importance" is defined as the most critical role in a specific response: the answer would depend on the specific function being considered (e.g., NTS for baroreflex control, LC for fight-or-flight response).

Therefore, based on the broad perspective of integrating and coordinating adrenergic outflow, the paraventricular nucleus (PVN) is the most important nucleus originating from the adrenergic CNS pathway.

However, it's important to remember that the adrenergic system is highly interconnected and involves complex interplay between various nuclei. The "importance" of any one nucleus can vary depending on the specific context and physiological scenario.

Please let me know if you have any further questions or if you'd like to explore the importance of the adrenergic pathway in a specific context.

**2- Imagine you are an expert physiologist with a specialization in neurophysiology. Answer the following question. {If there are three neurons A, B, and C connected such that neuron A connects to B and neuron B connects to C, and if neuron A is glutamatergic, neuron B is GABAergic, and neuron C is enkephalinergic. When neuron A is stimulated, will the signal transmitted from neuron B to neuron C be EPSP or IPSP?}. Explain the steps and reasons that lead you to the answer. write your final answer. let's think step by step.**

Step-by-step analysis of signal transmission:

1. Neuron A (glutamatergic) stimulates neuron B (GABAergic):

- Glutamate, released by neuron A, binds to NMDA and AMPA receptors on neuron B's membrane.
- This binding opens ion channels, allowing Na+ and K+ influx, leading to membrane depolarization.
- Depolarization triggers an action potential in neuron B.

2. Neuron B (GABAergic) releases GABA onto neuron C (enkephalinergic):

- GABA released by neuron B binds to GABA receptors (mainly GABAA) on neuron C's membrane.
- GABAA receptors are Cl- channels, meaning they allow Cl- influx upon binding GABA.
- Cl- influx hyperpolarizes the membrane of neuron C, reducing its excitability.

3. Conclusion:

Therefore, the signal transmitted from neuron B to neuron C will be an inhibitory postsynaptic potential (IPSP). This is because GABA released by neuron B hyperpolarizes neuron C, making it less likely to fire an action potential.

Additional notes:

- The specific type of enkephalin released by neuron C wouldn't directly affect the type of signal transmitted between B and C, although it could have downstream effects depending on the target neurons and receptors.
- The strength of the IPSP would depend on various factors, including the number and type of GABA receptors on neuron C, the amount of GABA released by neuron B, and the membrane potential of neuron C at the time of transmission.

I hope this step-by-step explanation clarifies the reasoning behind the answer. Please let me know if you have any further questions!

**3- Imagine you are an expert physiologist with a specialization in neurophysiology. Answer the following question. {Stimulation of which adrenergic receptor mainly has a regulatory role in presynaptic release? A) Alpha 1 B) Alpha 2 C) Beta 1 D) Beta 2 }. Explain the steps and reasons that lead you to the answer. write your final answer. let's think step by step.**

Thinking step-by-step:

1. Presynaptic release regulation:

We're looking for the adrenergic receptor primarily involved in regulating neurotransmitter release from the presynaptic neuron. This rules out beta receptors, which mainly act on postsynaptic neurons to alter their response to the released neurotransmitter.

2. Adrenergic receptor types:

Now we focus on alpha receptors. Alpha receptors are further divided into subtypes, each with distinct roles:

- Alpha 1: primarily located on smooth muscle cells, affecting blood pressure and smooth muscle contraction.
- Alpha 2: involved in various functions, including presynaptic modulation of neurotransmitter release.

3. Presynaptic alpha receptors:

Both alpha 2A and alpha 2C subtypes can be located presynaptically. However, their effects differ:

- Alpha 2A: primarily found on noradrenergic neurons, mediating a negative feedback loop where released norepinephrine inhibits further release through presynaptic alpha 2A receptors.
- Alpha 2C: has a broader distribution and can enhance or inhibit release depending on the neuron type and context.

4. Conclusion:

Based on the specific focus on presynaptic regulation of release, especially in the context of norepinephrine feedback inhibition, the key player emerges: Alpha 2A adrenergic receptor.

Therefore, the answer is B) Alpha 2.

Additional reason:

Stimulation of alpha 1 receptors generally leads to excitation or contraction, not inhibition, further supporting the exclusion of A) Alpha 1.

I hope this step-by-step explanation clarifies the reasoning behind the answer. Please let me know if you have any further questions!

**4- Imagine you are an expert physiologist with a specialization in neurophysiology. Answer the following question. {Is the LTP mechanism mostly mediated by glutamatergic pathways?}. Explain the steps and reasons that lead you to the answer. write your final answer. let's think step by step.**

Step 1: Defining the Key Terms

- LTP (Long-Term Potentiation): A fundamental process in learning and memory, where repeated high-frequency stimulation of a synapse strengthens it over a long period.
- Glutamatergic pathways: Neural signaling pathways that use glutamate as their primary neurotransmitter.

Step 2: Analyzing LTP Induction Mechanisms

- N-methyl-D-aspartate (NMDA) receptors: Key players in LTP induction. They allow calcium influx into postsynaptic neurons only upon receiving both glutamate and depolarization.
- AMPA receptors: Another type of glutamate receptor involved in LTP. They mediate the initial fast excitatory response at the synapse.
- Calcium influx: Increased calcium triggers downstream signaling cascades that lead to LTP-related changes, like protein phosphorylation and altered AMPA receptor trafficking.

Step 3: Examining the Evidence

- NMDA receptor blockade: Blocking NMDA receptors prevents LTP induction, highlighting their crucial role.
- AMPA receptor modulation: Manipulating AMPA receptor activity can influence LTP, but their role is secondary to NMDA receptors.
- Glutamate release: LTP induction protocols typically involve high-frequency stimulation, enhancing glutamate release from presynaptic neurons.
- Non-glutamatergic pathways: While some LTP induction can occur independently of glutamate, they are less common and often require specific conditions.

Step 4: Drawing Conclusions

Based on the evidence, we can conclude that:

- NMDA receptors and glutamate signaling play a pivotal role in LTP induction.
- Glutamate release and calcium influx triggered by NMDA receptor activation are crucial steps in LTP.
- Non-glutamatergic pathways can contribute to LTP under specific circumstances, but their contribution is significantly smaller.

Final Answer:

Yes, the LTP mechanism is primarily mediated by glutamatergic pathways, with NMDA receptors and glutamate signaling playing the central roles. While alternative pathways exist, their contribution is secondary and less prevalent.

Additional Notes:

- The specific LTP-inducing protocols and brain regions can influence the relative contribution of glutamatergic and non-glutamatergic mechanisms.
- Ongoing research continues to delve deeper into the complex and multifaceted nature of LTP.

Hopefully, this step-by-step approach provides a clear understanding of the reasoning behind the final answer. If you have any further questions or would like to delve deeper into specific aspects of LTP or neurophysiology, I'm happy to assist!

**5- Imagine you are an expert physiologist with a specialization in neurophysiology. Answer the following question. {Is myelination of postganglionic sympathetic fibers done by Schwann cells?}. Explain the steps and reasons that lead you to the answer. write your final answer. let's think step by step.**

Step-by-step analysis to determine the myelination of postganglionic sympathetic fibers:

1. Sympathetic Nervous System and Myelination:

- The autonomic nervous system (ANS) has two main branches: sympathetic and parasympathetic.
- The sympathetic nervous system is responsible for the "fight-or-flight" response and generally uses rapid signaling to organs.

2. Preganglionic and Postganglionic Neurons:

- The ANS pathway involves two sets of neurons:
  - Preganglionic neurons originate in the spinal cord and are myelinated.
  - Postganglionic neurons reside in ganglia closer to target organs and are typically unmyelinated.

3. Schwann Cells and Myelination:

- Schwann cells are the glial cells responsible for myelinating axons in the peripheral nervous system.
- They wrap around axons in a spiral, forming an insulating layer that speeds up action potential conduction.

4. Myelination in the Sympathetic System:

- The fast transmission required for the fight-or-flight response would seem to argue for myelination of postganglionic sympathetic fibers.
- However, research shows that these fibers are generally unmyelinated. This is likely due to:
  - Energy conservation: Myelination requires more energy, and the widespread distribution of sympathetic fibers makes it metabolically efficient to leave them unmyelinated.
  - Slower response in target organs: While rapid signaling to ganglia is crucial, the downstream effects on organs like smooth muscle and glands can often be slower and don't necessitate the high conduction speed afforded by myelin.

5. Exceptions to the Rule:

- Some specialized postganglionic sympathetic fibers, particularly those innervating sweat glands, can be partially myelinated. This might be due to the need for faster and more precise activation in these specific target organs.

Conclusion:

Based on the typical anatomy and functions of the sympathetic nervous system, and the general role of Schwann cells in peripheral myelination, postganglionic sympathetic fibers are not myelinated by Schwann cells. However, there are some rare exceptions.

Therefore, my final answer is no, Schwann cells do not typically myelinate postganglionic sympathetic fibers.

I hope this step-by-step explanation provides a clear and comprehensive understanding of the reasoning behind this answer. Feel free to ask any further questions you might have!

**6- Imagine you are an expert physiologist with a specialization in neurophysiology. Answer the following question. {Is the sensation of cold, like acute pain and unlike heat sensation, transmitted through myelinated Aδ (A delta) fibers?}. Explain the steps and reasons that lead you to the answer. write your final answer. let's think step by step.**

Step-by-step analysis:

1. Sensory receptor activation:

- Cold, like other temperature sensations and pain, is detected by specialized receptor cells called thermoreceptors in the skin.
- These thermoreceptors fall into two main categories:
  - Warm receptors respond to increasing temperatures.
  - Cold receptors respond to decreasing temperatures.

2. Signal transmission to the spinal cord:

- Once activated, thermoreceptors send electrical signals along sensory nerve fibers.
- Different types of nerve fibers carry different types of sensory information:
  - Group A fibers: These are myelinated fibers, meaning they have an insulating sheath around their axons, allowing for faster signal transmission.
  - Group C fibers: These are unmyelinated, slower-conducting fibers.

3. Specific nerve fiber involvement:

- Now, let's consider the options:
  - Heat sensation: Primarily transmitted by unmyelinated C fibers.
  - Acute pain: Transmitted by both myelinated Aδ fibers and unmyelinated C fibers.
  - Cold sensation: This is where it gets interesting.

4. Evidence for Aδ fiber involvement in cold sensation:

- Research indicates that Aδ fibers play a significant role in transmitting cold signals:
  - Physiological findings: Aδ fibers exhibit sensitivity to cooling stimuli similar to cold receptors.
  - Lesion studies: Damage to Aδ fibers can lead to impaired cold sensation, while C fiber damage primarily affects warmth.
  - Pharmacological experiments: Activation of Aδ fibers can mimic the sensation of cold.

5. Additional considerations:

- The role of C fibers in cold sensation cannot be completely ruled out. Some studies suggest they may contribute to a slower, sustained perception of cold.
- Individual differences in nerve fiber distribution and sensitivity can also play a role.

6. Conclusion:

Based on the available evidence, it is strongly suggested that the sensation of cold, like acute pain and unlike heat sensation, is primarily transmitted through myelinated Aδ fibers.

Remember: This is a complex area of research, and ongoing studies continue to refine our understanding of temperature and pain signaling pathways.

**7- Imagine you are an expert physiologist with a specialization in neurophysiology. Answer the following question. {Are sexual sensations mostly transmitted through the posterior column - medial lemniscus?}. Explain the steps and reasons that lead you to the answer. write your final answer. let's think step by step.**

Step 1: Identify the sensory modality of sexual sensations

Sexual sensations are a complex and multifaceted sensory experience that encompasses a range of sensations, including touch, pressure, temperature, and vibration. These sensations are primarily mediated by the somatosensory system, which is responsible for processing sensory information from the skin, muscles, and joints.

Step 2: Understand the organization of the somatosensory system

The somatosensory system is divided into two main pathways: the dorsal column-medial lemniscal pathway (DCML) and the anterolateral system (ALS). The DCML is responsible for transmitting fine touch, proprioception (body awareness), and vibration sensations. The ALS, on the other hand, is responsible for transmitting crude touch, pain, temperature, and itch sensations.

Step 3: Evaluate the role of the DCML in transmitting sexual sensations

The DCML is well-suited for transmitting fine touch and proprioceptive information due to its high degree of spatial resolution and sensitivity. However, sexual sensations also involve a significant component of pain, warmth, and cold sensations, which are typically transmitted through the ALS. Additionally, sexual sensations often arise from deep, internal organs, which are not directly innervated by the DCML.

Step 4: Consider the role of the ALS in transmitting sexual sensations

The ALS is capable of transmitting a wider range of sensory modalities than the DCML, including pain, warmth, cold, crude touch, and itch sensations. This makes it better suited for transmitting the full spectrum of sensations that contribute to sexual experiences. Additionally, the ALS has direct connections to the brainstem and limbic system, which are involved in processing emotions and arousal.

Step 5: Reach a conclusion

Based on the above evidence, it is more likely that sexual sensations are transmitted through the anterolateral system (ALS) rather than the posterior column-medial lemniscal pathway (DCML). The ALS is better equipped to handle the variety of sensory modalities involved in sexual experiences, and it has direct connections to the brainstem and limbic system, which are crucial for processing emotions and arousal.

Final answer:

No, sexual sensations are not mostly transmitted through the posterior column-medial lemniscus. They are more likely to be transmitted through the anterolateral system, which is better suited for transmitting the full range of sensations that contribute to sexual experiences.

**8- Imagine you are an expert physiologist with a specialization in neurophysiology. Answer the following question. {State key components, including nuclei and neurotransmitters, in the central nervous system analgesic pathway}. Explain the steps and reasons that lead you to the answer. write your final answer. let's think step by step.**

Step 1: Understanding pain sensation

Pain is a complex sensation that arises from the activation of nociceptors, specialized nerve cells that detect potentially harmful stimuli. These nociceptors transmit pain signals through ascending pathways to the central nervous system (CNS), where they are processed and interpreted.

Step 2: Identifying the descending pain modulation pathway

Alongside the ascending pain pathways, the CNS also possesses a descending pain modulation pathway that acts to inhibit pain transmission. This pathway utilizes a network of interconnected brain structures, including:

1. Periaqueductal gray (PAG): Located in the midbrain, the PAG serves as a central hub for pain modulation. It receives input from various sources, including the cortex, thalamus, and spinal cord, and sends descending signals to modulate pain transmission.
2. Locus coeruleus (LC): Situated in the pons, the LC plays a role in regulating arousal and stress responses. It releases norepinephrine, which can modulate pain transmission by influencing the activity of nociceptors and pain-transmitting neurons in the spinal cord.
3. Nucleus raphe magnus (NRM): Located in the medulla oblongata, the NRM is the primary source of descending pain modulation. It releases serotonin, which exerts potent analgesic effects by inhibiting pain transmission at various levels of the nervous system.

Step 3: Roles of neurotransmitters

Specific neurotransmitters play crucial roles in the descending pain modulation pathway:

1. Endorphins and endocannabinoids: These endogenous analgesics are released by the PAG and NRM, binding to opioid receptors to inhibit pain transmission.
2. Serotonin: As mentioned earlier, serotonin released from the NRM exerts strong analgesic effects by inhibiting the activity of pain-transmitting neurons.
3. GABA: Gamma-aminobutyric acid (GABA) is an inhibitory neurotransmitter that plays a role in overall pain modulation. It is released from various brain structures, including the PAG and periaqueductal gray, and inhibits the activity of pain pathways.

Step 4: Final answer

In summary, the key components of the central nervous system analgesic pathway include:

- Periaqueductal gray (PAG): Central hub for pain modulation
- Locus coeruleus (LC): Regulates arousal and releases norepinephrine for pain modulation
- Nucleus raphe magnus (NRM): Primary source of descending pain modulation, releases serotonin for strong analgesic effects
- Endorphins and endocannabinoids: Endogenous analgesics released by the PAG and NRM
- Serotonin: Inhibits pain transmission by binding to opioid receptors
- GABA: Inhibitory neurotransmitter that modulates overall pain perception

These structures and neurotransmitters work in concert to inhibit pain transmission, alleviating discomfort and promoting pain relief. Understanding the intricate mechanisms of the descending pain modulation pathway is crucial for developing effective pain management strategies.

**9- Imagine you are an expert physiologist with a specialization in neurophysiology. Answer the following question. {Which sensation is not transmitted through the anterolateral pathway? A) Chronic pain B) Cold sensation C) Touch sensation from Meissner receptor D) Touch sensation from Ruffini receptors}. Explain the steps and reasons that lead you to the answer. write your final answer. let's think step by step.**

Step 1: Identify the Anterolateral Pathway

The anterolateral pathway, also known as the ventral spinothalamic tract (VLST), is a major ascending sensory pathway that carries information about pain, temperature, and crude touch from the body to the brain. It is a contralateral pathway, meaning that the information from the left side of the body travels to the right side of the brain and vice versa.

Step 2: Analyze the Sensory Components

A) Chronic pain: Chronic pain is a persistent pain that lasts beyond the normal healing time for an injury. It is typically transmitted through the anterolateral pathway, as it is a type of pain that is important for survival.

B) Cold sensation: Cold sensation is also transmitted through the anterolateral pathway, as it is a vital component of our body's temperature regulation system.

C) Touch sensation from Meissner receptor: Meissner receptors are specialized touch receptors found in the skin that are responsible for detecting fine touch and vibration. Their signals are transmitted through the dorsal column-medial lemniscus pathway (DCML), not the anterolateral pathway.

D) Touch sensation from Ruffini receptors: Ruffini receptors are specialized touch receptors found in the skin that are responsible for detecting skin pressure and stretch. Their signals are also transmitted through the DCML pathway, not the anterolateral pathway.

Step 3: Determine the Correct Answer

Based on the analysis of the sensory components, the only sensation that is not transmitted through the anterolateral pathway is C) Touch sensation from Meissner receptor. The Meissner receptors, responsible for fine touch and vibration, send their signals through the DCML pathway, while the anterolateral pathway solely conveys pain, temperature, and crude touch information.

Final Answer:

The sensation that is not transmitted through the anterolateral pathway is touch sensation from Meissner receptors.

**10- Imagine you are an expert physiologist with a specialization in neurophysiology. Answer the following question. {Cortical areas related to sensations transmitted through which sensory pathways have a more precise and extensive topographical map? A) Posterior column - medial lemniscus pathway B) Paleospinothalamic pathway C) Anterior pathway D) Lateral pathway }. Explain the steps and reasons that lead you to the answer. write your final answer. let's think step by step.**

1. Identify the key feature: The question is asking about precise and extensive topographical maps in the cortex. This relates to the organization of sensory information based on its origin on the body.

2. Analyze the answer choices:

- A) Posterior column - medial lemniscus pathway (PCML): This pathway carries fine touch, vibration, and proprioception information. It is known to have a very precise and detailed topographical map in the primary somatosensory cortex (S1). Each point on the body has a corresponding point on S1, with greater detail given to areas with high touch sensitivity (e.g., fingertips).
- B) Paleospinothalamic pathway: Carries pain and temperature information. Its cortical map is less precise and extensive compared to the PCML.
- C) Anterior pathway: This term isn't commonly used in sensory pathways. It might be referring to the spinothalamic tract, which also carries pain and temperature information with a less precise map than the PCML.
- D) Lateral pathway: This likely refers to the lateral spinothalamic tract, carrying pain and temperature information. Similar to the paleospinothalamic pathway, its cortical map is less precise than the PCML.

3. Conclusion:

Based on the analysis, the posterior column - medial lemniscus pathway (PCML) is the only option with a well-established, highly precise, and extensive topographical map in the primary somatosensory cortex.

Therefore, the answer is A) Posterior column - medial lemniscus pathway.

Additional note:

- The terms "posterior column" and "medial lemniscus" can be used interchangeably to refer to the same pathway.
- Other sensory pathways, like the visual pathway, also have topographical maps in their respective cortical areas, but the question specifically asks about touch, vibration, and proprioception, which are conveyed by the PCML.

I hope this step-by-step explanation helps! Feel free to ask further questions if you'd like to delve deeper into any specific aspect.

**11- Imagine you are an expert physiologist with a specialization in neurophysiology. Answer the following question. {Can pontine reticular nucleus receive excitatory signals from the cerebellar fastigial nucleus?}. Explain the steps and reasons that lead you to the answer. write your final answer. let's think step by step.**

Step 1: Analyzing the potential connection:

a) Fastigial nucleus (FN):

- Located within the cerebellum, plays a crucial role in motor control and coordination.
- Sends descending projections to various brainstem structures, including the reticular formation.

b) Pontine reticular nucleus (PRN):

- Located within the pons, a component of the reticular formation.
- Involved in diverse functions like movement control, muscle tone, and autonomic regulation.

Based on their locations and known projections, a direct excitatory connection from FN to PRN is not likely. FN projections predominantly descend to the medulla, while PRN receives most of its excitatory input from the cerebral cortex and other pontine nuclei.

Step 2: Considering alternative pathways:

a) Indirect pathway:

- FN projects to the vestibular nuclei in the medulla, which then project to the PRN.
- This indirect pathway could potentially transmit excitatory signals from FN to PRN.

b) Modulatory effects:

- FN modulates PRN activity through non-direct mechanisms like influencing other brainstem nuclei or neurotransmitter release.

Step 3: Weighing evidence and drawing conclusions:

- Existing literature primarily focuses on FN's inhibitory influence on PRN activity through its projections to the medullary reticular formation and vestibular nuclei.
- Evidence for direct or indirect excitatory projections is scarce and requires further investigation.

Final Answer:

Based on current knowledge, it is unlikely that the pontine reticular nucleus receives direct excitatory signals from the cerebellar fastigial nucleus. However, an indirect excitatory pathway via the vestibular nuclei is a possibility, and non-direct modulatory effects are likely. Further research is needed to definitively confirm or refute the existence of direct excitatory projections.

Additional Notes:

- This answer considers current scientific understanding as of December 15, 2023. New research findings may emerge in the future.
- Neuroanatomy can be complex and nuanced, with intricate connections and interplays between different brain areas. Studying these connections requires sophisticated techniques and ongoing research efforts.

I hope this step-by-step explanation has been helpful! Please let me know if you have any further questions.

**12- Imagine you are an expert physiologist with a specialization in neurophysiology. Answer the following question. {Compared to the vermis, does the cerebellar intermedial area have a greater role in movement precision?}. Explain the steps and reasons that lead you to the answer. write your final answer. let's think step by step.**

Step-by-step analysis of the cerebellum's role in movement precision:

1. Function of the cerebellum:

- The cerebellum plays a crucial role in refining and coordinating motor commands, contributing to movement accuracy, precision, and timing.

2. Cerebellar anatomy:

- The cerebellum is divided into several regions, each with distinct functions:
  - Vermis: Located midline, it processes information related to balance, gait, and posture.
  - Intermediate area: Located laterally, it's involved in planning and coordinating limb movements.

3. Specific roles of vermis and intermedial area in movement:

- Vermis:
  - Maintains body posture and equilibrium by integrating sensory inputs from eyes, ears, and proprioceptors.
  - Less direct influence on fine limb control and precise movements.
- Intermedial area:
  - Receives extensive inputs from motor cortex, brainstem, and spinal cord.
  - Processes these inputs to fine-tune motor commands for limb trajectories, muscle activation, and force modulation.
  - Plays a critical role in skilled movements requiring high precision and accuracy.

4. Evidence for the intermedial area's role in movement precision:

- Studies using lesions, electrical stimulation, and neuroimaging have shown that damage to the intermedial area leads to:
  - Tremors and incoordination during limb movements.
  - Difficulty with reaching, grasping, and manipulating objects.
  - Impaired fine motor skills like writing or playing instruments.

5. Conclusion:

Based on the functional differentiation between vermis and intermedial area, combined with the evidence from neuroscience research, we can conclude that:

The cerebellar intermedial area has a greater role in movement precision compared to the vermis.

Reasons:

- The intermedial area directly processes information crucial for planning and executing precise limb movements.
- Vermis focuses more on global motor control and stabilization, with less direct influence on fine motor skills.
- Damage to the intermedial area disrupts skilled movements requiring high accuracy, whereas vermis lesions primarily affect balance and posture.

This is not to say the vermis has no role in movement precision. Both regions contribute, but with distinct specialties. The vermis ensures a stable platform for movement, while the intermedial area fine-tunes the muscle actions for accurate and coordinated execution.

Please remember that this is a simplified explanation. The cerebellum is a complex structure with ongoing research uncovering new details about its intricate functions.

**13- Imagine you are an expert physiologist with a specialization in neurophysiology. Answer the following question. {Is hypersexuality seen in Kluver-Bucy syndrome?}. Explain the steps and reasons that lead you to the answer. write your final answer. let's think step by step.**

Analyzing Kluver-Bucy Syndrome and Hypersexuality

As a neurophysiologist specializing in behavior, let's delve into the question of hypersexuality in Kluver-Bucy syndrome (KBS). To reach a well-informed answer, we'll take a step-by-step approach:

1. Defining the terms:

- Kluver-Bucy syndrome (KBS): A rare neurological disorder characterized by damage to the temporal lobes, particularly the amygdala and hippocampus. Symptoms include hyperorality, visual agnosia, emotional blunting, and hypersexuality (in some cases).
- Hypersexuality: Increased preoccupation with or an excessive need for sexual activity.

2. Understanding the neural basis of sexual behavior:

- The limbic system, particularly the amygdala and hypothalamus, plays a crucial role in sexual motivation and behavior.
- The amygdala processes emotions and social cues, influencing sexual desire and arousal.
- The hypothalamus regulates hormones and sends signals to the body for physical responses to sexual stimuli.

3. Examining the effects of KBS on relevant brain regions:

- KBS-related damage to the amygdala can disrupt its regulation of emotions and social cues, potentially leading to disinhibition of sexual behavior.
- However, the extent and location of damage within the amygdala and other limbic structures can vary, influencing the expression of hypersexuality.

4. Considering alternative explanations:

- Hypersexuality in KBS may not solely arise from brain damage; factors like pre-existing sexual behaviors, medication side effects, and psychosocial influences can also contribute.

5. Weighing the evidence and reaching a conclusion:

- While hypersexuality is a recognized symptom in some cases of KBS, it's not invariably present in all patients.
- The complex interplay of brain damage, individual differences, and external factors makes it difficult to conclusively link KBS and hypersexuality in every case.

Therefore, based on the current understanding of KBS and its effects on the brain, a definitive answer cannot be given. Hypersexuality can occur in some cases, but individual variations and additional factors influence its presence and severity.

Remember, this is a complex topic with ongoing research. If you have specific concerns about KBS or hypersexuality, please consult a qualified healthcare professional for personalized advice and support.

I hope this step-by-step analysis provides a comprehensive understanding of the issue while adhering to safety and ethical guidelines.

**14- Imagine you are an expert physiologist with a specialization in neurophysiology. Answer the following question. {Does microinjection of glutamate into the medullary reticular nucleus cause relaxation of axial muscles?}. Explain the steps and reasons that lead you to the answer. write your final answer. let's think step by step.**

Step 1: Identify the function of the medullary reticular nucleus

The medullary reticular nucleus is a complex structure in the brainstem that contains a variety of neuron types. It plays a role in regulating many bodily functions, including:

- Motor control: The medullary reticular nucleus helps to control the activity of spinal cord motor neurons, which control the muscles of the body.
- Autonomic nervous system: The medullary reticular nucleus is also involved in regulating the autonomic nervous system, which controls involuntary functions such as heart rate, breathing, and digestion.
- Sensory processing: The medullary reticular nucleus receives sensory input from the body and helps to process and integrate this information.

Step 2: Determine the action of glutamate on the medullary reticular nucleus

Glutamate is a major excitatory neurotransmitter in the brain. It acts on a variety of different receptors, some of which are excitatory and some of which are inhibitory. In the medullary reticular nucleus, glutamate acts on a number of different receptors, including NMDA, AMPA, and kainate receptors. These receptors are involved in a variety of different processes, including:

- Neurotransmission: Glutamate is released from neurons in the medullary reticular nucleus and acts on receptors on other neurons in the nucleus. This process helps to coordinate the activity of different neurons in the nucleus.
- Synaptic plasticity: Glutamate can also induce long-term changes in the strength of synapses, which are the connections between neurons. These changes are important for learning and memory.

Step 3: Consider the potential effects of glutamate microinjection on axial muscle relaxation

Given the information above, we can make some inferences about the potential effects of glutamate microinjection on axial muscle relaxation.

- Excitation of motor neurons: Glutamate can excite motor neurons in the spinal cord, which can lead to increased muscle contraction. This suggests that glutamate microinjection into the medullary reticular nucleus could potentially cause contraction of axial muscles.
- Inhibition of inhibitory interneurons: Glutamate can also inhibit inhibitory interneurons in the spinal cord. This suggests that glutamate microinjection into the medullary reticular nucleus could potentially cause disinhibition of motor neurons, which could also lead to increased muscle contraction.

Step 4: Consider the overall balance of excitation and inhibition

The final effect of glutamate microinjection on axial muscle relaxation will depend on the overall balance of excitation and inhibition in the medullary reticular nucleus. If the excitatory effects of glutamate are greater than the inhibitory effects, then glutamate microinjection could lead to increased muscle contraction. However, if the inhibitory effects are greater than the excitatory effects, then glutamate microinjection could lead to decreased muscle contraction.

Step 5: Reach a conclusion

Based on the information above, it is possible that glutamate microinjection into the medullary reticular nucleus could cause relaxation of axial muscles. However, the specific effect would depend on a number of factors, including the dose of glutamate, the location of the injection, and the activity of other neurons in the nucleus.

Conclusion:

Further research is needed to determine the exact effects of glutamate microinjection on axial muscle relaxation. However, based on the current evidence, it is possible that glutamate microinjection could be a potential treatment for conditions such as spasticity.

**15- Imagine you are an expert physiologist with a specialization in neurophysiology. Answer the following question. {Does stimulation of paraventricular nuclei produce effects similar to amygdala stimulation?}. Explain the steps and reasons that lead you to the answer. write your final answer. let's think step by step.**

Step 1: Understanding the Paraventricular Nuclei (PVN)

The paraventricular nuclei (PVN) are a group of neurons located in the hypothalamus, the control center of the brain. They are involved in a wide range of physiological functions, including:

- Regulation of the autonomic nervous system
- Hormonal regulation
- Control of energy metabolism
- Behavioral responses to stress

The PVN is a complex structure, with over 20,000 neurons. These neurons project to many different brain regions, including the amygdala, the hippocampus, the prefrontal cortex, and the medulla oblongata.

Step 2: Understanding the Amygdala

The amygdala is a small almond-shaped structure located deep within the temporal lobe of the brain. It is part of the limbic system, which is involved in emotions, memory, and motivation. The amygdala is particularly important for processing fear and other negative emotions.

The amygdala has extensive connections with the PVN. These connections allow the amygdala to influence the PVN's activity, and vice versa.

Step 3: Comparing PVN and Amygdala Stimulation

Studies have shown that stimulation of the PVN and the amygdala can produce a wide range of effects, including:

- Increases in heart rate and blood pressure
- Elevated levels of stress hormones, such as cortisol
- Changes in behavioral responses to stress

However, the specific effects of stimulation can vary depending on the intensity and duration of the stimulation, as well as the individual animal's physiology and behavior.

Step 4: Analyzing Similarities and Differences

In general, stimulation of the PVN and the amygdala can have similar effects on the body, such as increased heart rate and blood pressure. This is because both structures are involved in the regulation of the autonomic nervous system.

However, there are also some important differences between the two structures. For example, the amygdala is more specifically involved in processing and responding to fear, while the PVN is more generally involved in regulating physiological functions.

Step 5: Synthesizing the Information

Based on the available evidence, it is clear that stimulation of the PVN and the amygdala can have some similar effects, particularly on the autonomic nervous system. However, there are also important differences between the two structures, reflecting their distinct roles in the brain.

Final Answer:

Stimulation of the paraventricular nuclei can produce effects similar to amygdala stimulation, but there are also important differences between the two structures. The PVN is involved in a wider range of physiological functions than the amygdala, which is more specifically involved in processing and responding to fear.

Conclusion:

The paraventricular nuclei and the amygdala are both important brain structures that play a role in regulating physiological and behavioral responses to stress. While they share some similarities, they also have distinct roles in the brain. Understanding these differences is important for understanding how the brain responds to stress and how it can be manipulated therapeutically.

**16- Imagine you are an expert physiologist with a specialization in neurophysiology. Answer the following question. {In medical science and neurophysiology, is knowing "my birthday is January 10, 1998" an example of semantic explicit memory?}. Explain the steps and reasons that lead you to the answer. write your final answer. let's think step by step.**

Step 1: Define Semantic Explicit Memory

Semantic explicit memory is a type of long-term memory that stores general knowledge about the world, such as facts, concepts, and vocabulary. It is a conscious and declarative type of memory, meaning that we can consciously access and declare the information stored in it.

Step 2: Identify the Information to Be Stored

The information to be stored in this case is the birthday date, "January 10, 1998." This is a specific fact about the person's life, and it is stored in the semantic memory system.

Step 3: Determine the Type of Memory

Semantic explicit memory is the most likely type of memory to store this information because it is a general fact about the world, and it is consciously accessible. Other types of memory, such as episodic memory, would be less likely to store this information because they are typically related to personal experiences that are not explicitly recalled.

Step 4: Verify the Answer

Based on the above analysis, knowing "my birthday is January 10, 1998" is an example of semantic explicit memory. This is because it is a general fact about the world that is consciously accessible.

**17- Imagine you are an expert physiologist with a specialization in neurophysiology. Answer the following question. {In dementia, neurons secreting which neurotransmitters are more destroyed}. Explain the steps and reasons that lead you to the answer. write your final answer. let's think step by step.**

Certainly, let's delve into the neurophysiology of dementia and identify the neurotransmitters most affected by this debilitating condition.

Step 1: Understand the role of neurotransmitters in brain function

Neurotransmitters are chemical messengers that facilitate communication between neurons, the basic units of the nervous system. They are released from the presynaptic neuron, cross the synaptic cleft, and bind to receptors on the postsynaptic neuron, triggering a cascade of events that ultimately influence the neuron's activity. Neurotransmitters play a crucial role in various brain functions, including memory, learning, mood, and movement.

Step 2: Examine the impact of dementia on neurotransmitter levels

Dementia is a general term for a decline in cognitive function severe enough to interfere with daily life. It is often caused by the progressive loss of neurons and their neurotransmitter-producing machinery. Studies have shown that dementia is associated with a decrease in the levels of certain neurotransmitters, including:

1. Acetylcholine: Acetylcholine is a neurotransmitter essential for memory, learning, and attention. Its deficiency is a hallmark of Alzheimer's disease, the most common form of dementia.
2. Dopamine: Dopamine is involved in movement, motivation, and reward. Its depletion is associated with Parkinson's disease, which is sometimes characterized by dementia.
3. GABA: GABA is an inhibitory neurotransmitter that regulates brain activity and promotes relaxation. Its deficiency can contribute to anxiety and insomnia, which are often associated with dementia.
4. Serotonin: Serotonin regulates mood, appetite, and sleep. Its dysregulation has been linked to depression and agitation, which are common symptoms of dementia.

Step 3: Identify the most affected neurotransmitters in dementia

While the specific neurotransmitters affected vary depending on the type of dementia, acetylcholine is consistently implicated in the majority of cases. This is due to the high vulnerability of cholinergic neurons in the hippocampus, a brain region crucial for memory formation and consolidation. The loss of these neurons leads to a dramatic decline in acetylcholine levels, which is a major factor in the memory impairments seen in dementia.

Step 4: Formulate the final answer

Based on the neurophysiological underpinnings of dementia, the most affected neurotransmitters are acetylcholine, dopamine, GABA, and serotonin. These neurotransmitters play critical roles in various brain functions, and their depletion contributes to the cognitive, behavioral, and psychological symptoms associated with dementia.

In summary, the destruction of neurons secreting acetylcholine, dopamine, GABA, and serotonin is a major contributing factor to the neurodegenerative process and the cognitive, behavioral, and psychological manifestations of dementia. Understanding these neurotransmitter imbalances provides valuable insights into the underlying mechanisms of this debilitating condition and the development of potential therapeutic interventions.

**18 -** **Imagine you are an expert physiologist with a specialization in neurophysiology. Answer the following question. {Do X cells have the highest abundance among retinal ganglion cells?}. Explain the steps and reasons that lead you to the answer. write your final answer. let's think step by step.**

Step 1: Identify the type of retinal ganglion cells (RGCs)

Before determining whether X cells have the highest abundance among RGCs, it's crucial to accurately identify the type of RGCs we're referring to. Retinal ganglion cells comprise a diverse population of neurons, each with distinct functional properties and anatomical characteristics. Identifying the specific type of X cell is essential for providing an accurate response.

Step 2: Review the abundance of different RGC types

Once the type of X cells is established, it's necessary to gather information on the relative abundance of different RGC subtypes. This information can be obtained from anatomical studies, physiological experiments, and clinical observations. By comparing the abundance of X cells to other RGC types, we can determine their relative frequency in the retina.

Step 3: Analyze the functional role of X cells

The abundance of a particular RGC type is often correlated with its functional role. For instance, RGCs that respond to high spatial acuity, such as parvocellular RGCs, are more abundant than those that respond to low spatial acuity, such as magnocellular RGCs. Understanding the functional role of X cells can provide insights into their relative abundance.

Step 4: Consider the evolutionary context

The distribution of RGC subtypes can also be influenced by evolutionary factors. For example, diurnal animals tend to have a higher proportion of RGCs that respond to high spatial acuity, while nocturnal animals have a higher proportion of RGCs that respond to low light conditions. Understanding the evolutionary history of X cells can shed light on their abundance.

Step 5: Integrate the information and reach a conclusion

By considering the abundance of different RGC types, their functional roles, the evolutionary context, and any available experimental data, we can reach a conclusion about whether X cells have the highest abundance among retinal ganglion cells.

Final Answer:

Without knowing the specific type of X cells, it is impossible to determine definitively whether they have the highest abundance among retinal ganglion cells. However, by considering the factors outlined above, one can reach a more informed conclusion. If X cells are a subtype of parvocellular RGCs, which are known for their high spatial acuity and are relatively abundant in the retina, then it is highly probable that X cells have a relatively high abundance among RGCs. However, if X cells belong to a different RGC subtype with a more specialized or restricted role, their abundance may be lower compared to other RGC types. Therefore, the answer to the question depends on the precise definition of "X cells."

**19- Imagine you are an expert physiologist with a specialization in neurophysiology. Answer the following question. {In medical science and neurophysiology, which of the following represents explicit memory? A) The Shahnameh is the masterpiece of the great Iranian poet named Ferdowsi B) Today I arrived about 7 minutes late to physiology class. C) In 2010 my house had a major fire D) One of my elementary school friends' lastname ended in "Abadi" or "Abadian". Explain the steps and reasons that lead you to the answer. write your final answer. let's think step by step.**

Step 1: Defining explicit memory

Explicit memory, also known as declarative memory, is a type of long-term memory that involves the conscious recollection of factual information and personal experiences. This type of memory is often associated with the ability to verbally or nonverbally declare or describe the information that is being recalled.

Step 2: Identifying explicit memory characteristics

Explicit memory is characterized by the following features:

1. Consciousness: Explicit memories are consciously accessible and can be intentionally recalled.
2. Factuality: Explicit memories can be about facts, events, or concepts, and they can be verbalized or expressed in other ways.
3. Semantic vs. Episodic: Explicit memory can be divided into two categories: semantic memory, which stores general knowledge and facts, and episodic memory, which stores specific personal experiences and events.

Step 3: Analyzing the provided options

Let's analyze the provided options in light of the characteristics of explicit memory:

Option A: The Shahnameh is the masterpiece of the great Iranian poet named Ferdowsi

Recalling the author and title of a literary masterpiece

This option involves recalling factual information about a literary work and its author. It is a piece of knowledge that can be consciously recalled and expressed verbally. Therefore, this option represents explicit memory.

Option B: Today I arrived about 7 minutes late to physiology class.

Recalling a recent personal event

This option involves recalling a specific personal experience, the time of arrival to physiology class. It is a memory of an event that happened recently and can be consciously recalled. Therefore, this option also represents explicit memory.

Option C: In 2010 my house had a major fire.

Recalling a past personal event

This option involves recalling a specific personal experience, a house fire that occurred in 2010. It is a memory of a past event that can be consciously recalled. Therefore, this option also represents explicit memory.

Option D: One of my elementary school friends' lastname ended in "Abadi" or "Abadian".

Recalling a vague detail of a past experience

This option involves recalling a partial and possibly vague detail of a past experience, the last name of an elementary school friend. It is a memory of a past event that is not as clear or specific as the other options. This level of detail may not fall under the strict definition of explicit memory, but it could be considered a weaker form of explicit memory or even a transition between explicit and implicit memory.

Step 4: Final answer

Based on the analysis of the provided options and the characteristics of explicit memory, the following options represent explicit memory:

A) The Shahnameh is the masterpiece of the great Iranian poet named Ferdowsi

C) In 2010 my house had a major fire

Option B, "Today I arrived about 7 minutes late to physiology class", also demonstrates explicit memory, as it involves recalling a recent personal event. Option D, "One of my elementary school friends' lastname ended in 'Abadi' or 'Abadian'," is a more ambiguous case, as it involves recalling a vague detail of a past experience. While this may not be considered a classic example of explicit memory, it could represent a weaker form of explicit memory or even a transition between explicit and implicit memory.

**20- Imagine you are an expert physiologist with a specialization in neurophysiology. Answer the following question. {Deficiency of which amino acid can cause sleep disruption? A) Trp B) Leu C) Ala D) Gly }. Explain the steps and reasons that lead you to the answer. write your final answer. let's think step by step.**

Step-by-step analysis to determine the amino acid causing sleep disruption:

1. Understanding the role of amino acids in sleep:

- Several amino acids play crucial roles in regulating sleep-wake cycles, primarily through their involvement in neurotransmitter synthesis.
- Neurotransmitters are chemical messengers that facilitate communication between neurons and influence various brain functions, including sleep and wakefulness.

2. Examining the options:

- Trp (Tryptophan): A precursor for the neurotransmitter serotonin and the hormone melatonin, both critical for sleep induction and maintenance. Low Trp levels can disrupt sleep regulation.
- Leu (Leucine): Primarily involved in protein synthesis and energy metabolism, with limited influence on sleep-related neurotransmitters.
- Ala (Alanine): Primarily involved in energy metabolism and gluconeogenesis, with no known direct involvement in sleep regulation.
- Gly (Glycine): Plays a role in several neurotransmitter systems, including glycine itself, which has inhibitory effects in the brain. However, its role in sleep regulation is less clear than Trp.

3. Reasoning and elimination:

- Exclude Leu, Ala, and Gly: Based on their primary functions and limited involvement in sleep-related neurotransmitters, Leu, Ala, and Gly are less likely candidates for causing sleep disruption due to deficiency.
- Focus on Trp: Trp stands out for its direct involvement in the synthesis of crucial sleep-regulating molecules like serotonin and melatonin. Deficiency in Trp can significantly limit the production of these sleep-inducing substances, leading to sleep problems.

4. Conclusion:

Therefore, based on the role of each amino acid in neurotransmitter synthesis and sleep regulation, the most likely amino acid whose deficiency can cause sleep disruption is:

A) Trp (Tryptophan)

Additional notes:

- This analysis provides a simplified explanation. Sleep regulation is a complex process involving many factors, and amino acid deficiencies are just one possible contributing factor.
- It is important to consult a healthcare professional if you experience persistent sleep problems for proper diagnosis and personalized treatment.

I hope this step-by-step explanation helps you understand the reasoning behind the answer! Feel free to ask any further questions you may have.

**Claude**

**1- Imagine you are an expert physiologist with a specialization in neurophysiology. Answer the following question. {What is the most important nucleus originating from the adrenergic CNS pathway?}. Explain the steps and reasons that lead you to the answer. write your final answer. let's think step by step.**

As an expert physiologist specialized in neurophysiology, here is my thought process to identify the most important nucleus originating from the adrenergic central nervous system (CNS) pathway:

The adrenergic pathways utilize norepinephrine as a neurotransmitter to activate adrenergic receptors and influence various functions like heart rate, blood pressure, arousal, cognition etc. These pathways originate from brainstem nuclei that synthesize and release norepinephrine.

The main adrenergic nuclei in the brainstem are:

1. The locus coeruleus
2. The lateral tegmental nuclei
3. The solitary nucleus

Of these, the locus coeruleus located in the pons is the largest group of norepinephrine-producing neurons. It sends adrenergic projections throughout the brain and spinal cord and is considered to play pivotal roles in arousal, attention, cognition and autonomic regulation.

Some key reasons why the locus coeruleus is the most important adrenergic nucleus:

- It is the primary source of norepinephrine release in the cortex and hippocampus, modulating higher cognitive functions
- It has widespread projections enabling it to influence whole-brain arousal and vigilance states
- Changes to the locus coeruleus have been implicated in neuropsychiatric conditions like depression and Alzheimer’s

In summary, based on its substantial norepinephrine-synthesizing neurons, broad projections and involvement in complex neural functions, the locus coeruleus stands out as the most important nucleus originating from the CNS adrenergic pathways.

Final Answer: The locus coeruleus

**2- Imagine you are an expert physiologist with a specialization in neurophysiology. Answer the following question. {If there are three neurons A, B, and C connected such that neuron A connects to B and neuron B connects to C, and if neuron A is glutamatergic, neuron B is GABAergic, and neuron C is enkephalinergic. When neuron A is stimulated, will the signal transmitted from neuron B to neuron C be EPSP or IPSP?}. Explain the steps and reasons that lead you to the answer. write your final answer. let's think step by step.**

Certainly, I would be happy to explain the neural signaling process in that three neuron pathway. As a physiologist specialized in neurophysiology, here is my step-by-step reasoning:

Step 1) Neuron A is glutamatergic, meaning it releases the excitatory neurotransmitter glutamate. When neuron A is stimulated, it will release glutamate onto neuron B.

Step 2) Neuron B is GABAergic, meaning it releases the inhibitory neurotransmitter GABA. When glutamate from neuron A binds receptors on neuron B, it will open ion channels and cause an excitatory postsynaptic potential (EPSP) in neuron B.

Step 3) The EPSP in neuron B will make it more likely to fire an action potential. When neuron B fires, it will release GABA onto neuron C.

Step 4) Neuron C is enkephalinergic, meaning it releases the inhibitory peptide neurotransmitter enkephalin. When GABA from neuron B binds receptors on neuron C, it will open chloride channels and cause an inhibitory postsynaptic potential (IPSP) in neuron C.

Conclusion) Therefore, when neuron A is stimulated, the signal transmitted from neuron B to neuron C will be an IPSP due to neuron B releasing the inhibitory neurotransmitter GABA onto neuron C.

In summary, the signal transmission is:

1. Stimulate glutamatergic neuron A
2. Neuron A releases glutamate, causing EPSP in GABAergic neuron B
3. Neuron B fires action potential, releasing GABA onto enkephalinergic neuron C
4. GABA from neuron B causes IPSP in neuron C

So the final signal from neuron B to neuron C is an inhibitory postsynaptic potential (IPSP).

**3- Imagine you are an expert physiologist with a specialization in neurophysiology. Answer the following question. {Stimulation of which adrenergic receptor mainly has a regulatory role in presynaptic release? A) Alpha 1 B) Alpha 2 C) Beta 1 D) Beta 2 }. Explain the steps and reasons that lead you to the answer. write your final answer. let's think step by step.**

As an expert physiologist specialized in neurophysiology, here is my thought process to answer this question:

First, I know that adrenergic receptors play important regulatory roles in neurotransmitter release from presynaptic nerve terminals. Specifically, alpha-2 adrenergic receptors provide inhibitory feedback to reduce further norepinephrine release.

Next, I analyze the answer choices systematically:

A) Alpha-1 receptors generally mediate excitatory post-synaptic actions rather than presynaptic inhibition when activated.

B) Alpha-2 receptors activate Gi proteins which inhibit adenylyl cyclase activity and reduce cAMP production. This provides negative feedback on vesicles containing neurotransmitters, reducing their further release presynaptically.

C) Beta-1 receptors mainly have chronotropic and inotropic effects on heart function. They do not directly regulate presynaptic neurotransmitter release.

D) Beta-2 receptors facilitate neurotransmitter release through Gs protein activation and increased cAMP. However, they typically have more metabolic and postsynaptic effects.

By process of elimination examining the specific effects of each adrenergic receptor subtype, I conclude that alpha-2 receptors are responsible for the key regulatory inhibitory mechanism on presynaptic neurotransmitter release.

Therefore, my final answer is B) Alpha 2

The key steps in my reasoning are:

1. Recalling alpha-2 receptor mediated inhibition of presynaptic neurotransmitter release
2. Systematically examining each answer option
3. Ruling out incorrect choices based on specific receptor physiological functions
4. Selecting alpha-2 receptors as the best answer through reasoning and elimination

**4- Imagine you are an expert physiologist with a specialization in neurophysiology. Answer the following question. {Is the LTP mechanism mostly mediated by glutamatergic pathways?}. Explain the steps and reasons that lead you to the answer. write your final answer. let's think step by step.**

As an expert physiologist specialized in neurophysiology, here is how I would approach answering whether long-term potentiation (LTP) is mostly mediated by glutamatergic pathways:

First, I would define LTP and glutamatergic pathways. LTP refers to the strengthening of synapses between neurons based on patterns of activity. It is considered one of the major cellular mechanisms underlying memory and learning. Glutamatergic pathways use the neurotransmitter glutamate and make up the major excitatory pathways in the brain.

Next, I would review the key evidence about the mechanisms underlying LTP:

- LTP relies on NMDA receptors, which are glutamate receptors, becoming activated and allowing calcium into the postsynaptic neuron when the presynaptic neuron releases glutamate. This calcium then triggers downstream changes that strengthen the synapse.
- Experiments blocking NMDA receptors prevent LTP, while activating NMDA receptors can induce LTP. This demonstrates their critical role.
- Other glutamate receptors like AMPA receptors are also crucial for LTP expression, undergoing changes in number and properties during LTP to enhance transmission.
- Animal models where glutamatergic signaling is disrupted show deficits in LTP and learning abilities.

Given the central role of both NMDA receptors and AMPA receptors, which bind glutamate and mediate excitatory post-synaptic potentials, this evidence clearly implicates glutamatergic neurotransmission in the induction and expression of LTP.

My conclusion is:

Yes, the LTP mechanism is mostly mediated by glutamatergic pathways. Glutamate neurotransmission, particularly via NMDA and AMPA receptors, is critical for producing the synaptic changes underlying long-term potentiation. Disrupting glutamatergic signaling prevents the induction and expression of LTP.

**5- Imagine you are an expert physiologist with a specialization in neurophysiology. Answer the following question. {Is myelination of postganglionic sympathetic fibers done by Schwann cells?}. Explain the steps and reasons that lead you to the answer. write your final answer. let's think step by step.**

As an expert physiologist specialized in neurophysiology, here is my thought process to answer whether myelination of postganglionic sympathetic fibers is done by Schwann cells:

Step 1) Recall that the sympathetic nervous system has preganglionic neurons that originate in the spinal cord and synapse with postganglionic neurons outside the spinal cord.

Step 2) Remember that preganglionic sympathetic fibers are myelinated while postganglionic sympathetic fibers are unmyelinated. Myelination speeds up the propagation of action potentials along axons.

Step 3) Review that myelination in the peripheral nervous system is carried out by Schwann cells. These glial cells wrap around axons and form the myelin sheath.

Step 4) Infer that since postganglionic sympathetic fibers are unmyelinated, they do not undergo myelination by Schwann cells. The lack of myelin allows slower, non-rapid signaling from the postganglionic neuron to the target tissue.

Final Answer: No, myelination of postganglionic sympathetic fibers is not done by Schwann cells because these fibers are unmyelinated. Myelination by Schwann cells only occurs around preganglionic sympathetic fibers to allow faster conduction velocities.

**6- Imagine you are an expert physiologist with a specialization in neurophysiology. Answer the following question. {Is the sensation of cold, like acute pain and unlike heat sensation, transmitted through myelinated Aδ (A delta) fibers?}. Explain the steps and reasons that lead you to the answer. write your final answer. let's think step by step.**

As an expert physiologist specialized in neurophysiology, here is my thought process to answer whether the sensation of cold, like acute pain and unlike heat sensation, is transmitted through myelinated Aδ fibers:

Step 1) Review what is known about Aδ fibers. Aδ fibers are lightly myelinated peripheral nerve fibers that transmit acute, well-localized fast pain and temperature sensations.

Step 2) Consider the neural pathways for temperature sensation. Temperature sensation is transmitted along small diameter fibers including unmyelinated C fibers (respond slowly to warm/cool stimuli) and myelinated Aδ fibers (respond quickly to noxious hot/cold stimuli).

Step 3) Reflect on the sensation of acute pain. Acute pain is transmitted rapidly via Aδ fibers to allow the body to react quickly to potentially damaging stimuli. This is an important survival mechanism.

Step 4) Think about the sensation of heat. Heat sensation is transmitted more slowly along unmyelinated C fibers. This allows us to detect non-damaging increases in temperature without setting off acute pain signals.

Step 5) Contemplate the sensation of cold. Like acute pain and unlike heat, the sensation of extreme cold is transmitted rapidly via Aδ fibers. This allows us to react quickly to potentially damaging decreases in temperature.

Final Answer: Yes, the sensation of cold, like acute pain and unlike heat sensation, is transmitted through myelinated Aδ (A delta) fibers. This allows for a rapid response to prevent tissue damage from extreme decreases in temperature. The Aδ fibers respond quickly to noxious cold stimuli, while heat sensations are transmitted more slowly along C fibers.

In summary, my expertise in neurophysiology leads me through the known functions of Aδ fibers, differences in temperature sensation pathways, how acute pain functions, and the purpose of rapid cold sensation. This step-by-step reasoning brings me to confirm that cold sensation does indeed utilize Aδ fibers to transmit signals quickly, similar to acute pain and unlike heat.

**7- Imagine you are an expert physiologist with a specialization in neurophysiology. Answer the following question. {Are sexual sensations mostly transmitted through the posterior column - medial lemniscus?}. Explain the steps and reasons that lead you to the answer. write your final answer. let's think step by step.**

As an expert physiologist specializing in neurophysiology, here is how I would approach answering whether sexual sensations are mostly transmitted through the posterior column-medial lemniscus pathway:

Step 1: Recall the relevant anatomy and physiology of the pathways that transmit sensations from the body to the brain. The posterior column-medial lemniscus pathway transmits fine touch, vibration sense, and proprioception from the body to the brain. A separate pathway called the anterolateral system transmits pain, temperature, and crude touch sensations.

Step 2: Consider what types of sensations are experienced during sexual activity. While there are some components of fine touch, sexual activity also involves significant sensations of temperature and pain/pleasure that are transmitted via the anterolateral system.

Step 3: Evaluate whether the posterior column pathway could transmit the majority of these diverse sensations on its own. Given the anatomy and functions of the two pathways, it is unlikely that complex sexual sensations are transmitted predominantly through the posterior column system alone.

Step 4: Infer which pathway is most important. The diversity of sensations experienced during sex, involving temperature, pain, and pleasure, suggest the anterolateral spinothalamic tract plays the major role in transmitting sexual sensations to the brain.

Final Answer: No, sexual sensations are not mostly transmitted through the posterior column-medial lemniscus pathway. The variety of sensations experienced during sexual activity, including temperature, pain, and pleasure, require the alternate anterolateral/spinothalamic tract to reach the brain. Therefore, this pathway transmits the majority of sexual sensations rather than the posterior column system.

**8-** **Imagine you are an expert physiologist with a specialization in neurophysiology. Answer the following question. {State key components, including nuclei and neurotransmitters, in the central nervous system analgesic pathway}. Explain the steps and reasons that lead you to the answer. write your final answer. let's think step by step.**

Certainly, I'd be happy to explain the central nervous system analgesic pathway. As an expert physiologist specializing in neurophysiology, here is how I would approach answering this question:

To start, I would focus my answer on key components of the descending analgesic pathway, since that is a major way that the central nervous system modulates pain perception. The key nuclei in this pathway are:

1. The periaqueductal gray (PAG) matter in the midbrain, which receives input from higher cortical regions related to pain perception and emotional response. The PAG then coordinates the descending analgesia response.
2. The PAG projects to the rostral ventromedial medulla (RVM) in the brainstem. The RVM directly communicates with the dorsal horn of the spinal cord to inhibit pain signaling transmission from the peripheral nociceptors.
3. The PAG and RVM both utilize opioid neurotransmitters like endorphins, enkephalins and dynorphins to exert their analgesic effects. These bind to opioid receptors on pain transmission neurons in the dorsal horn, causing hyperpolarization and reduced signaling to the brain.

In summary, higher brain regions activate the PAG in response to pain signals, initiating the release of endogenous opioid neurotransmitters. These act upon neurons in the RVM of the brainstem, which then directly communicates with the dorsal horn of the spinal cord to inhibit the transmission of peripheral pain signals to the brain. This creates a top-down analgesia effect mediated by opioids binding to inhibitory receptors on dorsal horn sensory neurons.

Final Answer: The key components in the central nervous system analgesic pathway are: the periaqueductal gray matter of the midbrain, the rostral ventromedial medulla of the brainstem, and endogenous opioid neurotransmitters like endorphins, enkephalins and dynorphins, which bind to opioid receptors on dorsal horn neurons to inhibit pain transmission.

**9- Imagine you are an expert physiologist with a specialization in neurophysiology. Answer the following question. {Which sensation is not transmitted through the anterolateral pathway? A) Chronic pain B) Cold sensation C) Touch sensation from Meissner receptor D) Touch sensation from Ruffini receptors}. Explain the steps and reasons that lead you to the answer. write your final answer. let's think step by step.**

Okay, let's go through this step-by-step:

The anterolateral pathway (ALP) transmits several different sensory modalities from the body to the brain. We need to determine which of the answer choices is NOT transmitted by this pathway.

First, let's review the sensations that ARE transmitted by the ALP:

- Pain and temperature sensory fibers travel in the ALP. So both chronic pain sensations and cold sensations would be transmitted by this pathway.

Now let's examine the answer choices: A) Chronic pain - Transmitted by ALP, so not the answer B) Cold sensation - Also transmitted by ALP, not the answer C) Touch sensation from Meissner receptors - Meissner receptors detect light touch. This would travel in the dorsal column medial lemniscal pathway, NOT the ALP.
D) Touch sensation from Ruffini receptors - Ruffini receptors detect stretch/pressure. This also goes in the DCML pathway rather than ALP.

So the correct answer must be either C or D, as touch sensations do not travel in the anterolateral pathway. Ruffini receptors detect stretch/pressure, while Meissner receptors detect light touch. But either way, the touch sensory fibers from these receptors travel in the dorsal column pathway rather than the ALP.

Therefore, the sensation that is NOT transmitted through the anterolateral pathway is: C) Touch sensation from Meissner receptors

**10- Imagine you are an expert physiologist with a specialization in neurophysiology. Answer the following question. {Cortical areas related to sensations transmitted through which sensory pathways have a more precise and extensive topographical map? A) Posterior column - medial lemniscus pathway B) Paleospinothalamic pathway C) Anterior pathway D) Lateral pathway }. Explain the steps and reasons that lead you to the answer. write your final answer. let's think step by step.**

As an expert physiologist specialized in neurophysiology, here is my thought process to answer this question:

The question is asking to compare the topographical mapping precision and extent among different sensory pathways that transmit sensations to the cortex. The options given are:

A) Posterior column - medial lemniscus pathway B) Paleospinothalamic pathway C) Anterior pathway D) Lateral pathway

First, I know that the posterior column-medial lemniscus pathway transmits fine touch, vibration sense, and proprioception from the body to the cortex. This pathway has very precise and extensive topographical mapping in the primary somatosensory cortex (S1).

Next, the paleospinothalamic pathway transmits crude touch and pressure sensations. While it projects to the cortex, the topographical mapping is less precise compared to the posterior column system.

The anterior pathway refers to the anterior spinothalamic tract, which carries pain and temperature. It has overlapping projections thus less extensive mapping.

Finally, the lateral pathway is likely referring to the lateral spinothalamic tract, also involved in pain and temperature sensations. This also has overlapping projections and less precision.

Based on this analysis, the posterior column-medial lemniscus pathway has the most precise and extensive topographical mapping among these pathways.

Therefore, my final answer is: A) Posterior column - medial lemniscus pathway

The precise mapping allows discrete touch sensations to be localized by the brain based on which part of the skin is stimulated. This is key for fine tactile discrimination.

**11- Imagine you are an expert physiologist with a specialization in neurophysiology. Answer the following question. {Can pontine reticular nucleus receive excitatory signals from the cerebellar fastigial nucleus?}. Explain the steps and reasons that lead you to the answer. write your final answer. let's think step by step.**

Certainly, I would be happy to analyze this neurophysiology question in detail. As an expert physiologist specialized in neurophysiology, here is my thought process:

To determine if the pontine reticular nucleus can receive excitatory signals from the cerebellar fastigial nucleus, I would systematically go through the relevant neuroanatomy and neurophysiology.

First, I know that the pontine reticular nucleus is located within the pons, which relays signals between the cerebellum and higher brain centers. The cerebellar fastigial nucleus projects to several subcortical structures.

Next, I would analyze if there are known anatomical connections between these two structures that could facilitate signal transmission. Tracing studies in animals have demonstrated that the cerebellar fastigial nucleus sends excitatory glutamatergic projections to the pontine reticular nucleus. This provides an anatomical pathway for signals to be transmitted.

I would then consider the physiology - excitatory signals typically work through glutamate receptors, including NMDA and AMPA receptors. Studies have shown that pontine reticular nucleus neurons express these types of glutamate receptors and are activated by glutamate agonists. This provides receptors for fastigial nucleus glutamatergic signals to activate pontine reticular neurons.

Finally, electrophysiology recordings have demonstrated that electrical stimulation of the fastigial nucleus drives action potential firing in pontine reticular nucleus neurons. This definitively demonstrates the pontine reticular nucleus can receive excitatory drive from the fastigial nucleus.

In conclusion, based on the neuroanatomy showing connections, receptor physiology, and electrophysiology recordings, I can definitively state that the pontine reticular nucleus can receive excitatory signals from the cerebellar fastigial nucleus. The anatomical and physiological evidence supports signal transmission between these structures.

**12- Imagine you are an expert physiologist with a specialization in neurophysiology. Answer the following question. {Compared to the vermis, does the cerebellar intermedial area have a greater role in movement precision?}. Explain the steps and reasons that lead you to the answer. write your final answer. let's think step by step.**

As an expert physiologist specializing in neurophysiology, here is my thought process to answer whether the cerebellar intermedial area has a greater role in movement precision compared to the vermis:

Step 1) Review what is known about the functions of the vermis and cerebellar intermedial area. The vermis is located in the median (midline) region of the cerebellum. It is involved in basic motor control and balance. The cerebellar intermedial area is located between the vermis and cerebellar hemispheres. Research shows it plays an important role in precise limb movements and motor learning.

Step 2) Consider the type of motor control and movement each region is more specialized for. The vermis is important for general, gross motor control related to balance and posture. In contrast, the cerebellar intermedial area seems to be more involved in fine-tuned control of limb movements requiring precision.

Step 3) Evaluate studies comparing damage to these areas. Lesions in the vermis lead to issues with trunk control, standing, and falling. But damage to the intermedial area causes more specific problems with accurately reaching targets with the limbs. This supports the intermedial area playing a greater role in precise motions.

Final Answer: Yes, based on location, connections, and lesion evidence, the cerebellar intermedial area has a greater specialized role in precise motor control and movement accuracy compared to the vermis. The vermis is more focused on gross motor skills like balance, while intermedial damage leads to specific deficits in limb precision.

In summary, by reviewing what is known about the functions of each region, and comparing their involvement in different types of movements, I can logically conclude the cerebellar intermedial area is more integral for precision than the vermis. My specialized expertise in neurophysiology allows me to systematically evaluate the evidence to determine this.

**13- Imagine you are an expert physiologist with a specialization in neurophysiology. Answer the following question. {Is hypersexuality seen in Kluver-Bucy syndrome?}. Explain the steps and reasons that lead you to the answer. write your final answer. let's think step by step.**

As an expert physiologist specialized in neurophysiology, here is how I would approach answering whether hypersexuality is seen in Kluver-Bucy syndrome:

First, I would review what Kluver-Bucy syndrome is. Kluver-Bucy syndrome involves a range of behavioral changes, including docility, placidity, hyperorality, hypersexuality, visual agnosia, and memory deficits. It is caused by bilateral lesions of the medial temporal lobe, including the amygdala and hippocampus.

Next, I would specifically focus my analysis on whether hypersexuality is one of the documented symptoms. In my knowledge and review of the literature on Kluver-Bucy syndrome, hypersexuality is indeed commonly seen. Patients exhibit inappropriate sexual behaviors like genital manipulation or excessive sexuality towards inappropriate objects or people.

The mechanisms behind this are not fully clear, but the medial temporal lobe regions are important in processing emotions and drives, including sexual drives. Damage to this area appears to lead to disinhibited and exaggerated sexual behaviors. The amygdala in particular is thought to regulate sexual behavior.

So in summary:

1. Kluver-Bucy syndrome involves behavioral changes due to bilateral medial temporal lobe lesions
2. One cardinal symptom is hypersexuality i.e. inappropriate or excessive sexual behaviors
3. This is thought to be caused by disinhibition of sexual drives due to amygdalar and related medial temporal lobe damage

Therefore, my expert conclusion as a neurophysiologist is that yes, hypersexuality can be seen in Kluver-Bucy syndrome.

**14- Imagine you are an expert physiologist with a specialization in neurophysiology. Answer the following question. {Does microinjection of glutamate into the medullary reticular nucleus cause relaxation of axial muscles?}. Explain the steps and reasons that lead you to the answer. write your final answer. let's think step by step.**

As an expert physiologist specializing in neurophysiology, here is my thought process to answer whether microinjection of glutamate into the medullary reticular nucleus causes relaxation of axial muscles:

First, I would consider the functions of the main components involved - glutamate and the medullary reticular nucleus. Glutamate is the main excitatory neurotransmitter in the central nervous system. When glutamate is microinjected into an area of the brain, it excites and activates that region.

The medullary reticular nucleus is part of the reticular formation in the brainstem. This area helps control axial muscles involved in posture, locomotion and other basic body movements. The neurons in the medullary reticular nucleus use glutamate as well as GABA and glycine as neurotransmitters.

Next, I would review any research that has been done injecting glutamate specifically into the medullary reticular nucleus and measuring effects on axial muscle tone. Glutamate would excite neurons in this nucleus that are involved in facilitating muscle contraction. Activating them would lead me to hypothesize that glutamate injection would cause contraction, not relaxation, of the axial muscles under control of the medullary reticular nucleus.

Finally, I would experimentally microinject glutamate into the medullary reticular nucleus of an animal model while monitoring effects on axial muscle tone through electromyography. Based on my background knowledge, I would expect to observe increased muscle contraction, validating my original hypothesis.

In summary, microinjection of the excitatory neurotransmitter glutamate into the medullary reticular nucleus involved in facilitating axial muscle contraction would lead me to hypothesize and experimentally confirm that this causes axial muscle contraction, not relaxation.

Therefore, my answer is no, microinjection of glutamate into the medullary reticular nucleus does not cause relaxation of axial muscles. It causes contraction by exciting neurons in this area.

**15- Imagine you are an expert physiologist with a specialization in neurophysiology. Answer the following question. {Does stimulation of paraventricular nuclei produce effects similar to amygdala stimulation?}. Explain the steps and reasons that lead you to the answer. write your final answer. let's think step by step.**

Certainly, I would be happy to analyze this neurophysiology question in detail. As an expert physiologist specializing in neurophysiology, here is how I would approach comparing the effects of paraventricular nucleus (PVN) stimulation versus amygdala stimulation:

To begin, I would consider the locations and functions of each brain region. The PVN is located in the hypothalamus and is involved in the regulation of autonomic functions as well as the release of hormones from the pituitary gland. In contrast, the amygdala is an almond-shaped structure deep in the temporal lobe that plays key roles in emotional processing and memory consolidation.

Next, I would systematically review research studies examining the impacts of electrically or chemically stimulating each region. Key effects of PVN stimulation include increases in heart rate, blood pressure, glucocorticoid release, as well as triggering of the fight-or-flight stress response. Amygdala stimulation has been found to produce fear and aggression behaviors, increased anxiety, altered social interactions, and facilitated emotional memory formation.

Comparing these effects side-by-side, there are some overlaps but also clear differences. Both PVN and amygdala stimulation can elicit increases in heart rate and blood pressure, likely via activation of the sympathetic nervous system. However, the PVN has much broader effects on endocrine function and the stress response axis. The amygdala seems to have more specific effects related to emotional behaviors and memories.

Therefore, based on this analysis, I would conclude that PVN and amygdala stimulation produce some similar effects on cardiovascular parameters, but differ in many other functional outcomes. The PVN is more involved in systemic stress responses, while the amygdala is more specialized for emotional and social processing. But there are certainly interactive links between the hypothalamic and limbic circuits.

In summary, no, PVN stimulation does not produce all the same effects as amygdala stimulation, though some effects may overlap. The differences relate to the distinct roles of the PVN and amygdala in coordinating physiological stress responses versus emotional behaviors. My expert neurophysiology evaluation leads me to this conclusion.

**16- Imagine you are an expert physiologist with a specialization in neurophysiology. Answer the following question. {In medical science and neurophysiology, is knowing "my birthday is January 10, 1998" an example of semantic explicit memory?}. Explain the steps and reasons that lead you to the answer. write your final answer. let's think step by step.**

As an expert physiologist specializing in neurophysiology, here is how I would approach determining if "knowing my birthday is January 10, 1998" is an example of semantic explicit memory:

Step 1: Define semantic memory and explicit memory

- Semantic memory refers to general factual knowledge about the world that is not tied to personal experiences
- Explicit (or declarative) memory refers to conscious memories that can be intentionally recalled and verbalized

Step 2: Analyze the statement "My birthday is January 10, 1998" contains a piece of factual personal information. While it is a fact, it is directly tied to an autobiographical personal experience rather than general factual knowledge.

Step 3: Semantic versus episodic explicit memory

- Explicit memory can be further categorized as either semantic (facts) or episodic (personal experiences)
- Knowing one's own birthday is an episodic memory about a personal experience rather than a semantic memory about a fact.

Final Answer: No, "knowing my birthday is January 10, 1998" is not an example of semantic explicit memory. It is an episodic explicit autobiographical memory about a personal life event. Semantic explicit memories contain factual information not tied to personal experiences.

In summary, the key steps were:

1. Define differences between memory types
2. Analyze if the statement contained general facts or personal experiences
3. Categorize if it fits semantic or episodic explicit memory definitions

**17- Imagine you are an expert physiologist with a specialization in neurophysiology. Answer the following question. {In dementia, neurons secreting which neurotransmitters are more destroyed}. Explain the steps and reasons that lead you to the answer. write your final answer. let's think step by step.**

As an expert physiologist specializing in neurophysiology, here is how I would approach answering which neurotransmitters are more destroyed in dementia:

First, I would reflect on my knowledge of the pathological hallmarks of different types of dementia. Alzheimer's disease, the most common cause of dementia, is characterized by buildup of amyloid plaques and neurofibrillary tangles composed of tau protein. This leads to neuronal dysfunction and death over time.

Next, I would consider which brain regions and neurotransmitter systems are most impacted in Alzheimer's disease. The key regions affected are the hippocampus, entorhinal cortex, and basal forebrain - areas critical for memory and cognition. These regions utilize acetylcholine as a major neurotransmitter. Research shows that in Alzheimer's, cholinergic neurons in these areas degenerate, resulting in reduced acetylcholine.

Other neurotransmitters like dopamine, serotonin, and norepinephrine also show deficits but the loss of cholinergic neurons is most pronounced. This aligns with the memory and cognitive symptoms that are hallmarks of Alzheimer's dementia.

Therefore, based on my expertise, I would conclude that in dementia, specifically Alzheimer's disease, neurons secreting the neurotransmitter acetylcholine are more severely destroyed. This leads to reduced cholinergic neurotransmission and the buildup of amyloid and tau pathological changes, contributing to the cognitive decline seen in Alzheimer's dementia.

In summary, acetylcholine-releasing neurons are more impacted in dementia, especially Alzheimer's disease, relative to other neurotransmitter systems. The loss of cholinergic innervation underlies key memory and cognitive symptoms. This explains why Alzheimer's treatments often aim to boost acetylcholine levels to compensate for neuronal degeneration.

**18 -** **Imagine you are an expert physiologist with a specialization in neurophysiology. Answer the following question. {Do X cells have the highest abundance among retinal ganglion cells?}. Explain the steps and reasons that lead you to the answer. write your final answer. let's think step by step.**

Certainly, I would be happy to analyze this question and provide a detailed explanation. As an expert physiologist specialized in neurophysiology, here is how I would approach answering whether X cells have the highest abundance among retinal ganglion cells:

Step 1: Review my knowledge of retinal ganglion cells. There are several main types - X cells, Y cells, and W cells. They have different physiological properties and functional roles in visual processing.

Step 2: Consider what is meant by "highest abundance". This seems to refer to whether X cells make up the largest percentage of total retinal ganglion cells.

Step 3: Consult anatomical research on abundances of different retinal ganglion cell types. Studies show that X cells make up around 70-80% of the total retinal ganglion cell population in cats and monkeys.

Step 4: Compare to abundances of other cell types. Y cells comprise around 10-15% of the total. W cells make up the remainder.

Step 5: Conclude that among the retinal ganglion cells, X cells do indeed have the highest total abundance across species studied so far. Their predominance matches their role in critical visual functions like motion detection.

Final Answer: Yes, X cells have the highest abundance among retinal ganglion cells, making up approximately 70-80% of the total retinal ganglion cell population. Their high numbers correspond to their functional importance in visual processing tasks like motion and pattern detection.

In summary, I leveraged my expertise in retinal physiology, parsed the key question, reviewed current scientific data on retinal cell abundances, compared among cell types, and used evidence-based reasoning to conclude that X cells are the most abundant ganglion cell type. Let me know if you need any clarification or have additional questions!

**19- Imagine you are an expert physiologist with a specialization in neurophysiology. Answer the following question. {In medical science and neurophysiology, which of the following represents explicit memory? A) The Shahnameh is the masterpiece of the great Iranian poet named Ferdowsi B) Today I arrived about 7 minutes late to physiology class. C) In 2010 my house had a major fire D) One of my elementary school friends' last name ended in "Abadi" or "Abadian"}. Explain the steps and reasons that lead you to the answer. write your final answer. let's think step by step.**

As an expert physiologist specializing in neurophysiology, here is how I would approach answering this multiple choice question about types of memory:

Explicit memory, also known as declarative memory, refers to the conscious, intentional recollection of factual information, previous experiences and concepts. It can be further divided into semantic memory (facts) and episodic memory (personal experiences).

To determine which options represent explicit/declarative memory, I would systematically evaluate each one:

A) The Shahnameh option provides a fact about a poetic work. Knowing facts and concepts is associated with semantic memory, a type of explicit memory.

B) The option about arriving late to class describes a personal episodic memory about an event I experienced. Episodic memory is another component of explicit memory.

C) The house fire option is also an episodic memory of an autobiographical event that occurred in my past. This is also representative of explicit memory.

D) Remembering an elementary school friend's last name relies on the semantic memory system that stores factual information. So this option reflects another aspect of explicit memory.

In summary, options A, B, C and D all involve recollection of prior knowledge or experiences, indicating they tap into explicit memory capacities mediated by the medial temporal lobe and prefrontal cortical regions of the brain.

Therefore, my final answer is that options A, B, C and D all represent examples of explicit memory. The capacity to consciously bring back facts, concepts and events from one's past reflects the activation of declarative memory systems.

**20- Imagine you are an expert physiologist with a specialization in neurophysiology. Answer the following question. {Deficiency of which amino acid can cause sleep disruption? A) Trp B) Leu C) Ala D) Gly }. Explain the steps and reasons that lead you to the answer. write your final answer. let's think step by step.**

As an expert physiologist specialized in neurophysiology, here is my thought process to answer this question:

First, I would consider which amino acid is a precursor to important neurotransmitters and neurohormones that regulate sleep-wake cycles and sleep. The amino acid tryptophan (Trp) comes to mind as it is a precursor for serotonin and melatonin.

Serotonin is an important neurotransmitter that promotes wakefulness. Melatonin is an important sleep hormone that helps regulate circadian rhythms and induce sleepiness.

Next, I would reason that a deficiency in tryptophan could disrupt production of these important chemicals and lead to sleep disturbances. Lack of serotonin during wakefulness could cause daytime drowsiness and fatigue. Lack of melatonin could make it harder to fall and stay asleep at night.

The other answer options - leucine, alanine and glycine - do not have known roles as precursors to neurotransmitters or hormones that directly impact sleep. Maybe if they caused some other essential body function to be disrupted severely it could secondarily influence sleep, but tryptophan has the most direct, primary effect.

Therefore, based on tryptophan's role in synthesizing serotonin and melatonin, and the importance of these chemicals for proper sleep-wake regulation, I would conclude that deficiency of tryptophan (Trp) can most directly cause sleep disruption.

Final Answer: A (Trp)
